# Contrasting viral infection strategies for single cell and colonial *Microcystis* populations consistent with Black Queen dynamics

**DOI:** 10.1093/ismejo/wraf244

**Published:** 2025-11-03

**Authors:** Xuhui Huang, Emily E Chase, Brittany N Zepernick, Robbie M Martin, Lauren E Krausfeldt, Helena L Pound, Hanqi Wu, Zheng Zheng, Steven W Wilhelm

**Affiliations:** Department of Environmental Science and Engineering, Fudan University, Shanghai 200438, PR China; Department of Microbiology, The University of Tennessee-Knoxville, Knoxville, TN 37996-0845, United States; Department of Microbiology, The University of Tennessee-Knoxville, Knoxville, TN 37996-0845, United States; Department of Microbiology, The University of Tennessee-Knoxville, Knoxville, TN 37996-0845, United States; Department of Microbiology, The University of Tennessee-Knoxville, Knoxville, TN 37996-0845, United States; Department of Microbiology, The University of Tennessee-Knoxville, Knoxville, TN 37996-0845, United States; Department of Microbiology, The University of Tennessee-Knoxville, Knoxville, TN 37996-0845, United States; Department of Environmental Science and Engineering, Fudan University, Shanghai 200438, PR China; Department of Environmental Science and Engineering, Fudan University, Shanghai 200438, PR China; Department of Microbiology, The University of Tennessee-Knoxville, Knoxville, TN 37996-0845, United States

**Keywords:** metatranscriptomics, harmful algal blooms, phage, infection

## Abstract

Cyanobacterial blooms dominated by *Microcystis* spp. pose significant ecological challenges, including the release of toxins and disruption of aquatic food webs. Although *Microcystis* can exist as free-living single cells or within dense mucilaginous colonies, the drivers and consequences of colony formation remain unclear. Here, we integrated metatranscriptomic datasets from two *Microcystis* bloom events in Lake Taihu, China, to analyze and to support findings on the functional differences between colonial and single-cell *Microcystis*. Our results confirmed colony expression profiles were disproportionately enriched in *Microcystis* transcripts compared to other prokaryotic taxa. This pattern exhibits Black Queen-like dynamics, where *Microcystis* assumes greater metabolic and defensive roles while associated bacteria reduce their transcriptional activity. Concomitantly, viral infection strategies diverged by *Microcystis* community morphology: colony-associated cells expressed lysogeny-associated genes, whereas single cells exhibited increased signatures of lytic infection. These data are consistent with the hypothesis that *Microcystis* colonies foster conditions favorable to lysogen formation—likely due to local high cell densities and the resulting advantage of superinfection immunity—whereas solitary cells experience stronger lytic pressure. On a broader scale, our findings refine the understanding of bloom dynamics by identifying how community morphological states coincide with distinct host–virus interactions. Cumulatively, this work underscores the importance of colony formation in shaping *Microcystis* ecology and highlights the need for further mechanistic studies to disentangle the complex interplay between phage infection modes, colony formation, and microbial community structure.

## Introduction

Cyanobacteria are among the most ecologically significant microorganisms in fresh waters, driving biogeochemical cycles that include carbon fixation and nutrient regulation. Yet, concomitantly with these benefits come considerable environmental challenges through the formation of harmful algal blooms [1]. *Microcystis* has emerged globally as a key player [2] due to its adaptability to environmental conditions [3]. Although colony formation is a hallmark of *Microcystis* in natural systems, this capability is often lost under laboratory conditions [4], underscoring the potential importance of environmental pressures in driving this phenomenon.

The formation of *Microcystis* colonies has been suggested to confer numerous ecological advantages, including resistance to chemical stress [5], high-light conditions [6], grazing pressure [7], facilitating buoyancy, and nutrient acquisition [6]. Central to many of these processes is the interaction between *Microcystis* and associated heterotrophic bacteria (i.e., microbiome or phycosphere community), which can be embedded within the extracellular polymeric substance (EPS) matrix surrounding colonies [8]. This phycosphere community, comprising diverse taxa, is hypothesized to play pivotal roles in nutrient cycling, organic matter decomposition, and resource exchange, creating a microenvironment that supports *Microcystis* proliferation and dominance in eutrophic waters [9].

Bacteria and phages represent the most abundant and genetically diverse entities on Earth, with phages often outnumbering their bacterial hosts by an order of magnitude [10]. Among them, many phages employ a dual strategy of infection: entering lytic or lysogenic cycles [10]. In the lysogenic state, phages integrate into the bacterial genome as prophages, establishing a symbiotic relationship that may impose a fitness cost by disrupting host gene expression but also potentially conferring adaptive advantages, such as regulating host gene expression [11], introducing or altering functions [10], facilitating bacterial DNA transfer [12], shaping bacterial communities [13–15], and conferring resistance to further phage infection via superinfection exclusion. These phage-mediated interactions are thought to drive significant functional and evolutionary shifts in bacterial hosts, particularly under environmental stress [16, 17].

In this study, we used 20- and 0.45-*μ*m-pore-size filters to enrich colonial and single-cell *Microcystis* bloom samples in Lake Taihu (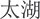 in Mandarin), China, and analyzed metatranscriptomic data to investigate the interplay between colony formation, the associated microbiome and viral infection. Additionally, we used data from another year to support the consistency of our observation. Previously we had hypothesized that lysogen formation or growth at high cell densities would be more prevalent when cyanobacterial blooms were at high densities [18]: we thought resistance to superinfection commonly seen in other lysogens could protect these massive blooms from collective lysis due to the high contact rates conditions would create [19]. By profiling the expression of lysogeny- and lytic-cycle-associated genes in a natural system, we demonstrated a positive correlation between colony formation and potential lysogen formation. However, the mechanism driving this phenomenon remains unclear—does lysogeny promotes colony formation by modulating host functions or does colony formation provide an environment conducive to lysogenic interactions? Our findings highlight the complexity of *Microcystis* ecological strategies and underscore the need for further research, including controlled experiments in laboratory settings, to disentangle the mechanistic links between lysogeny, colony formation, and microbial interactions.

## Materials and methods

### Sample collection

Surface water samples of a *Microcystis* bloom were collected from four sites (Supplementary Table 1) in Zhushan Bay, Lake Taihu on 26 August 2023 at midday. The separation of single cells and colonies of *Microcystis* was completed by a vacuum filter device (pressure range: 0 to −1 MPa) equipped with 20 *μ*m filter membranes. Subsequently filtrates were pass through 0.45 *μ*m filters to collect single cells. At each of four locations, sampling was performed in triplicate, resulting in a total of 12 colonial samples and 12 single-cell samples. Immediately after separation, the filter membranes were stored at −80°C until further processing. The particle size distribution of the colonial samples was measured using a laser particle size analyzer (BT-9300ST, Bettersize Instruments, Dandong, China) to confirm the presence of *Microcystis* colonies (Supplementary Fig. 1).

In parallel with the above samples, we accessed previously processed data collected and sequenced in 2018. Those six colonial and six single-cell samples were collected from the boat dock of the “Taihu Laboratory for Lake Ecosystem Research”*.* Samples were collected using 28 *μ*m mesh-size Nytex™ filtration material: retentate was maintained for the “colonial” size class and material passing through the filter was recollected onto a 0.2 *μ*m nominal pore-size polycarbonate filter mounted in a Swinnex holder and delivered with a sterile 60 CC syringe. Sample collections for the 2018 dataset are detailed in Supplementary Information.

### RNA extraction and sequencing

RNA samples were extracted and sequenced at Shanghai Majorbio Bio-pharm Biotechnology Co., Ltd. (Shanghai, China). Total RNA was extracted from the tissue using Soil RNA Extraction Kit (Majorbio, China). Total RNA was processed using the Illumina Stranded mRNA Prep, Ligation kit (Illumina, San Diego, CA, USA), with rRNA depletion performed using the RiboCop rRNA Depletion Kit for mixed bacterial samples (Lexogen, USA). Libraries were prepared following standard Illumina protocols and sequenced on a NovaSeq 6000 platform (Illumina; paired-end mode). Sample collection, RNA extraction and sequencing of 2018 samples are detailed in Supplementary Information.

### Pangenome assembly

To identify the transcriptomic differences between *Microcystis* morphotypes, 16 complete, closed *Microcystis* genomes (Supplementary Table 2) were downloaded from the National Center for Biotechnology Information (NCBI) to establish a *Microcystis* pangenome [20]. All individual genomes were merged into a single file and redundant coding sequences were subsequently removed via CD-HIT (nucleotide identity of 0.95) (v.4.8.1) [21]. Functional annotation of the *Microcystis* pangenome was derived from individual genomes, which were automatically annotated using NCBI Prokaryotic Genome Annotation Pipeline [22], additional annotations were supplemented using EggNOG-mapper (v.2.1.12) [23] using a specified e-value of 1e^−10^. Using the same pipeline, a *Microcystis* phage pangenome was constructed from 41 complete *Microcystis* phage genomes (Supplementary Table 3).

### Metatranscriptomic analysis

The sequence analysis of both 2023 and 2018 libraries used the following pipeline: bioinformatic trimming of raw reads was performed using fastp (v.0.23.2) [24]. Subsequently, reads were interleaved together using reformat.sh script available in the BBTools suite(v.38.18) [25]. Residual rRNA and contaminants were removed using the JGI reference database and bbmap.sh(v.38.18) [25] (minid = 0.93). The quality of reads was checked using FastQC (v.0.12.1) before and after trimming. Trimmed and filtered libraries (*n* = 24) were concatenated and assembled via MEGAHIT (v.1.2.9) [26], with quality of coassembly confirmed via QUAST QC (v.5.0.2) [27]. Read mappings were performed using bbmap.sh (v.38.18) [25] (minid = 0.90) to align mRNA reads to the coassembly, *Microcystis* pangenome and *Microcystis* phage pangenome, respectively. The summary of sequence information of 2023 libraries was listed in Supplementary Table 4. Read counts were tabulated by featureCounts (v.2.0.6) [28].

Gene predictions were performed using MetaGeneMark (v.3.38) [29] with the metagenome-style model. Taxonomic annotation of predicted genes was performed using Kraken 2 (v.2.1.3) [30] with the RefSeq complete genomes dataset (including archaea, bacteria, viral, fungi, and plant genomes). Functional annotations of predicted genes were supplemented using EggNOG-mapper (v.2.1.12) [23] using a specified e-value of 1e^−10^.

### Statistical analyses

Mapped reads were normalized to transcripts per million (TPM), representing relative transcript abundance, to account for differences in library size and gene length. The terms “overrepresented” and “underrepresented” are used to describe changes in the proportional representation of transcripts within the total transcriptome pool across conditions. All analyses were conducted in R [31] (v.4.4.2). For microbial relative abundance and gene expression levels, Shapiro–Wilk test was used to assess the normality of paired differences. As all comparisons met the normality assumption (*P* > .05), paired *t*-test was applied. Mean values are reported with standard deviation (SD). Normality of the log-transformed gene expression values from both 2018 and 2023 datasets was assessed using the Shapiro–Wilk test and did not meet the assumption of normality. Given the large number of genes analyzed, Pearson correlation was applied, with Spearman’s rank correlation used as a validation. Clustering of the normalized libraries was visualized via nonmetric multidimensional scaling (NMDS) using Bray–Curtis dissimilarity, implemented in the vegan (v.2.6-8) package. KEGG pathway enrichment analysis was performed using the clusterProfiler [32] (v.4.14.4) package. Heatmaps were generated with the pheatmap (v.1.0.12) package, and all additional figures were created using the ggplot2 [33] (v.3.5.1) package. Differential expression (DE) analysis was conducted using DESeq2 [34] (v.1.28.1), with genes showing a baseMean <10 excluded as noise. Genes with a log_2_ fold change >1 (log_2_|FC| >1) and an adjusted *P* value of <.05 were considered differentially expressed.

## Results

### Colonies are less taxonomically diverse than single-cell samples

**Figure 1:**
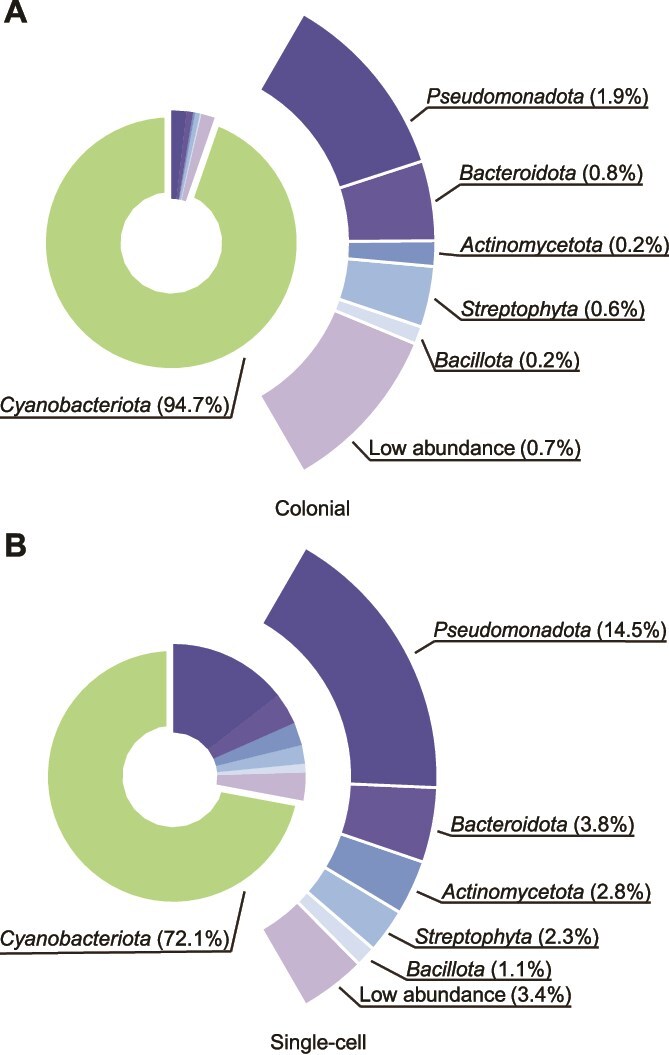
Microbiota of colonial and single-cell samples from the 2023 dataset. (A), (B) Phylum-level distribution of the microbiota in colonial samples (A, *n* = 12) and single-cell samples (B, *n* = 12) collected in 2023. *Cyanobacteriota* are represented in the inner donut chart. The outer ring highlights the relative abundances of non-*Cyanobacteriota* phyla, with corresponding percentages indicated beside the taxa.

We observed significant differences in the taxonomic composition between colonial (Fig. 1A) and single-cell (Fig. 1B) samples. *Cyanobacteriota* dominated both colonial and single-cell samples, accounting for an average of 94.7% ± 1.5% SD in colonial samples and 72.1% ± 10.5% SD in single-cell samples, with a significantly higher proportion in the colonial samples (*P* = .20, Shapiro–Wilk test; *P* < .001, paired *t*-test). Excluding *Cyanobacteriota*, the top five phyla which all demonstrated lower relative abundance in colonial samples compared with single-cell sample and included *Pseudomonadota* (*P* = .75, Shapiro–Wilk test; *P* < .001, paired *t*-test), *Bacteroidota* (*P* = .52, Shapiro–Wilk test; *P* < .001, paired *t*-test), *Actinomycetota* (*P* = .09, Shapiro–Wilk test; *P* < .001, paired *t*-test), *Streptophyta* (*P* = .10, Shapiro–Wilk test; *P* < .001, paired *t*-test) and *Bacillota* (*P* = .33, Shapiro–Wilk test; *P* < .001, paired *t*-test). At the genus level, *Microcystis* was the most abundant within the phylum *Cyanobacteriota*, accounting for an average of 91.7% ± 3.1% SD in colonial samples and 67.9% ± 9.6% SD in single-cell samples (Supplementary Fig. 2). During the 2023 sampling, *Microcystis* dominated both large colonies and small particles like what *Cyanobacteriota* did in phylum level, whereas other bacteria and eukaryotes were present at concentrations 1–2 orders of magnitude lower. These trends were also observed in 2018 samples (Supplementary Fig. 3).

### *Microcystis* dominates transcription of biological processes in colonies

**Figure 2:**
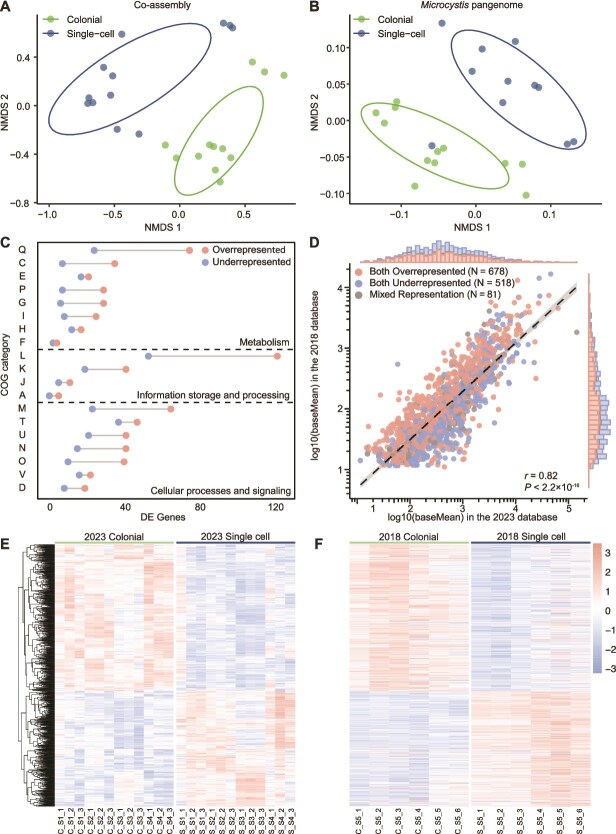
Functional distribution based on the co-assembly and *Microcystis* pangenome. (A), (B) NMDS plot based on Bray–Curtis dissimilarity of transcriptomic data from 2023 samples, mapped to the co-assembly (A) and the *Microcystis* pangenome (B), using TPM normalized data. Ellipses cover 68% of the data for each form. (C) Distribution of 2166 differentially expressed (DE) *Microcystis* genes across COG functional categories in the 2023 dataset. (D) Pearson correlation analysis of log10-transformed baseMean values for 1277 shared significant genes in the 2018 and 2023 datasets. (E), (F) Heatmap of 1277 shared significant genes across colonial and single-cell samples from the 2023 (E) and 2018 (F) dataset. Gene expression values were normalized to z-scores based on TPM values, and hierarchical clustering was performed on genes in the 2023 dataset, with the 2018 dataset using the same order. DE genes: differentially expressed genes.

Gene expression of both the microbiota and *Microcystis* differed in the colony samples relative to single-cell samples. The nMDS of Bray–Curtis dissimilarity based on gene expression from both the co-assembly (Fig. 2A) and *Microcystis* pangenome (Fig. 2B) were separately evaluated. DE analysis among the top six phyla revealed distinct transcriptional patterns (Table 1). In *Cyanobacteriota*, overrepresented and underrepresented genes between colonies and single cells were comparable. However, in other bacteria and eukaryotes, 97.8%–99.9% of the relevant expression of the genes were underrepresented in colonial samples. Within *Microcystis* alone, 81.6% of the genes in *Microcystis* increased expression in colonial libraries, whereas 97.8% of the genes in other *Cyanobacteriota* decreased expression. This latter pattern was similar to that of other bacteria and eukaryotes. The KEGG pathway enrichment analysis genes with decreased expression among all microbiota, excluding *Microcystis*, were primarily enriched in biosynthesis of cofactors and amino acids, and carbon metabolism (Supplementary Fig. 5). Within these same pathways, *Microcystis* showed more genes with increased expression (*n* = 212) in colonies than decreased expression (*n* = 116) (Supplementary Fig. 5).

**Table 1:** Number of DE genes based on the co-assembly and annotated by KEGG.

Taxonomy	DE genes	Overrepresented genes in colonies	Underrepresented genes in colonies
*Cyanobacteriota*	10 509	4423	6086
*Microcystis*	5404	4346	1058
*Cyanobacteriota* excluding *Microcystis*	5105	77	5028
*Pseudomonadota*	54 612	66	54 546
*Bacteroidota*	17 150	23	17 127
*Actinomycetota*	11 515	13	11 502
*Streptophyta*	4801	117	4684
*Bacillota*	2491	16	2474

To further investigate how community morphology contributed to the dissimilarity in *Microcystis* expression, DE gene analyses were performed based on the *Microcystis* pangenome and 2023 dataset. In total, 2166 genes belonging to *Microcystis* were differentially expressed (|Log_2_ FC| ≥ 1, *P_adj_* < .05), with 1547 of these genes increased in relative expression in colonial samples and 619 decreased. Across each COG category, most genes exhibited increased expression in colonial samples (Fig. 2C). Genes categorized in COG category L (Replication, recombination and repair, *n* = 175) were the most highly represented category based in the DE dataset, with 69.7% of them overrepresented in colonial samples. Of those genes, the majority belonged to transposase-encoding genes, with 64 identified, of which 52 (81.3%) showed decreased expression in colonial samples (Supplementary Fig. 7). Other genes associated with mobile genetic elements, such as genes encoding endonuclease and reverse transcriptase, also showed increased relative expression in colonies. Likewise, relative expression of genes within COG category Q (Secondary metabolites biosynthesis, transport and catabolism) increased in colonies (Supplementary Fig. 7). Other genes, including those encoding PEP-CTERM protein, gas vesicles and calcium-binding protein exhibited increased expression in colonial samples (Supplementary Fig. 7). Similar results were observed in 2018 samples (Supplementary Figs 6, 8).

To confirm our 2023 observations, a 2018 dataset of 12 libraries was also examined to find the DE genes in *Microcystis*. We identified 1277 shared genes (adjusted *P* < .05) present in both datasets (summary of these genes are provided in Supplementary Information), 93.7% of the genes showed consistent regulated results in two datasets. Correlation analysis (Fig. 2D) based on the average expression of shared genes in the two datasets revealed a strong positive correlation (Pearson correlation, *r* = .82, *P* < .001; Spearman’s rank correlation, *ρ* = .81, *P* < .001). We generated a gene clustering heatmap (Fig. 2E) using the 2023 dataset. Using the same gene order, we constructed a corresponding heatmap with the 2018 dataset (Fig. 2F). The results revealed consistent trends across both datasets, demonstrating that the significantly expressed genes exhibited the same regulatory patterns in both datasets. This indicated that the functional expression differences in *Microcystis* that are associated with differences in morphology were reproducible in Lake Taihu.

### Differential expression of genes from *Microcystis*-infecting phage

**Figure 3:**
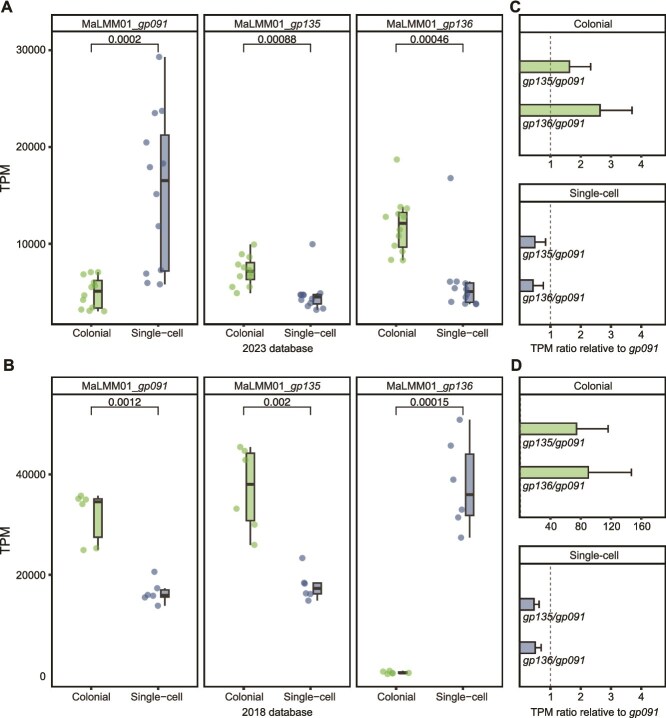
Relative expressions of lysogeny- and lysis-associated genes. (A), (B) TPM values of lysogeny- and lysis-associated genes in colonial and single-cell samples from the 2023 (A) and 2018 (B) dataset. *gp091* represents a marker for lytic infection, whereas *gp135* and *gp136* represent markers for lysogenic infection. The horizontal bars within the boxes represent medians, with the tops and bottoms of the boxes indicating the 75th and 25th percentiles, respectively. The upper and lower whiskers extend to the furthest data points within 1.5× the interquartile range from the edges of the box. (C), (D) TPM ratio of lysogeny-associated genes (*gp135* and *gp136*) relative to the lysis-associated gene (*gp091*) in colonial and single-cell samples from the 2023 (A) and 2018 (B) dataset. Ratios greater than 1 indicate lysogeny-dominant activity, whereas ratios less than 1 indicate lysis-dominant activity. Error bars represent the standard error of the mean for each ratio. The dashed vertical line at 1 represents equal dominance between lysogeny and lysis.

Based on *Microcystis* dominating both colonial and single-cell samples, as well as the functional differences driven by these morphological distinctions, we examined the expression of *Microcystis* phage genes to explore infection dynamics. Construction of the *Microcystis* phage pangenome revealed that six phages—Ma-LMM01, MaMV-DC, MaMV-DL01, MaMV-DL02, MaMV-CH01, and MaMV-CH02—clustered with each other, with pairwise similarity ranging from 60.1% to 100.0% (Supplementary Fig. 9A). After mapping sample reads to the phage pangenome, only these six Ma-LMM01-like phages and Mic1 were recruited, with the Ma-LMM01-like phages accounting for 83.5% ± 7.7% SD of the total mapped reads (Supplementary Fig. 9B). Ma-LMM01, with its fully sequenced genome, has served as an important model for studying *Microcystis*-phage interactions. Normalized expression of the Ma-LMM01 markers of lytic infection (tail sheath, *gp091*) [18], and putative lysogenic infection (transposase, *gp135*, and site-specific recombinase, *gp136*) [18] were observed in colonial and single-cell samples (Fig. 3A). Homologous genes clustered with *gp091* and *gp135* were also identified in MaMV-DC, MaMV-DL01, MaMV-DL02, MaMV-CH01, and MaMV-CH02 with high sequence similarity (99.6%–99.96% for gp091 and 99.1%–99.6% for gp135), while genes homologous to *gp136* were found in MaMV-DL01, MaMV-DL02, MaMV-CH01, and MaMV-CH02 (98.6% similarity). Of the 24 samples from the 2023 dataset, significant differences in infection strategies were observed. Colonial samples showed lower abundant expression of *gp091* (*P* = .25, Shapiro–Wilk test; *P* < .001, paired *t*-test) and higher abundant expression of *gp135* and *gp136* than single-cell samples (*P* = .19, .07, Shapiro–Wilk test; *P* < .001, paired *t*-test). In colonial samples, *gp135* (*P* = .20, Shapiro–Wilk test; *P* = .012, paired *t*-test) and *gp136* (*P* = .57, Shapiro–Wilk test; *P* < .001, paired *t*-test) exhibited significantly higher expression levels compared to *gp091* (Fig. 3C), with *gp135* showing a 1.64 ± 0.72 SD-fold increase and *gp136* displaying a 2.65 ± 1.08 SD-fold increase, implying that lysogenic infection was dominant. In single-cell samples, *gp091* exhibited significantly higher expression than *gp135* (*P* = .82, Shapiro–Wilk test; *P* = .002, paired *t*-test) and *gp136* (*P* = .91, Shapiro–Wilk test; *P* = .007, paired *t*-test), with *gp091* showing a 3.84 ± 2.58 SD-fold and 3.38 ± 2.27 SD-fold increase over *gp135* and *gp136*, respectively (Fig. 3C), implying those free single *Microcystis* cells experienced more lytic events. The expression of these maker genes was also examined in the 2018 dataset (Fig. 3B and D), revealing a consistent trend but with even greater difference. We also examined several additional putative markers of lytic and lysogenic infections in both datasets, which showed consistent expression trends (Supplementary Figs 10 and 11). These expression pattern differences are consistent across different collection years, lake locations, and times of day. For Mic1, nearly all of its genes were overrepresented in colonial samples. Mic1 may represent a subset of the viral community capable of adopting both lytic and lysogenic lifestyles [35], although the regulatory factors underlying these infection modes remain unclear.

## Discussion

This study focused on the dominant genus of cyanobacterial blooms in Lake Taihu, *Microcystis*, and explored its community dynamics, interactions with associated bacteria, and the infection strategies of *Microcystis* phages. Using metatranscriptomic data collected in 2023, we examined differences in species abundance and functional activity across colonial and single-cell forms of *Microcystis* and its associated microbes, as well as the contrasting infection strategies of *Microcystis* phages in these two forms. The findings in Lake Taihu were supported by comparisons with a similar 2018 metatranscriptomic dataset.

In each location, colonial and single-cell fractions were derived from the same original sample and therefore shared identical environmental parameters (Supplementary Table 1). This design minimizes the influence of external factors and supports the interpretation that observed differences in microbial taxonomic composition and functional potential are primarily driven by morphological form, rather than environmental variation. The differences are further likely driven by the dominant role of *Microcystis* within the colonies and its interactions with associated microbes.

Colonial *Microcystis* is often considered to be more competitive than the single-cell form due to enhanced resilience to environmental stresses provided by its structural advantages, such as the EPS matrix that offers physical protection and facilitates nutrient acquisition [6]. Colony formation is also thought to shield individual cells from environmental pressures [6]. However, overrepresentation of many other transcripts in colonial *Microcystis* suggests that significant metabolic investment is required to maintain the colony. This duality implies a trade-off: whereas colonies may offer greater defense and stability, from this work they appear to demand higher metabolic effort, which ultimately may shape the competitive fitness of colonial *Microcystis*.

Several specific gene groups may play key roles in colonial *Microcystis*. Genes encoding transposases were highly represented among the DE genes, this may reflect a rapid microevolutionary response under environmental stress, enhancing the adaptive flexibility of colonies [36]. Additionally, PEP-CTERM domain genes, previously shown to anchor exopolysaccharides and mediate cell aggregation in other bacteria [37], were also overrepresented in colonies, suggesting an essential role in forming and maintaining colony structure. Genes associated with gas vesicle formation similarly exhibited increased expression, consistent with their contribution to buoyancy control and light optimization [38]. But these expression data alone cannot resolve causality and warrant further experimental validation.

*Microcystis* genes exhibit increased expression in colonies, whereas the transcripts of associated microbes were reduced in representation. This suggests that these microbiome members need to maintain more active metabolic pathways in single-cell environments. We hypothesize that during the transition from single-cell to colonial *Microcystis*, microbial interactions undergo significant changes. In the initial stages of colony formation, microbes may collaborate but rely on their individual metabolic pathways. Once the colony is established, the system exhibits Black Queen-like dynamics [39]. In that context *Microcystis* assumes a central role, taking on the most metabolic and defensive burdens, whereas associated microbes downregulate gene expression, benefiting from a stable and protective environment where metabolic burdens are assumed by the cyanobacterium. It remains unclear if *Microcystis* suppresses the bacterial activity or whether the bacteria undergo self-inhibition. To this end, although some researchers have hypothesized that blooms form and persist due to the activity of associated microbial community members [40], it appears that the opposite may be in part true: blooms and particularly colonies provide a haven for many heterotrophic bacteria, perhaps through both the increased physical protection from the colony as well as the surplus of metabolic products *Microcystis* appears to produce.

**Figure 4:**
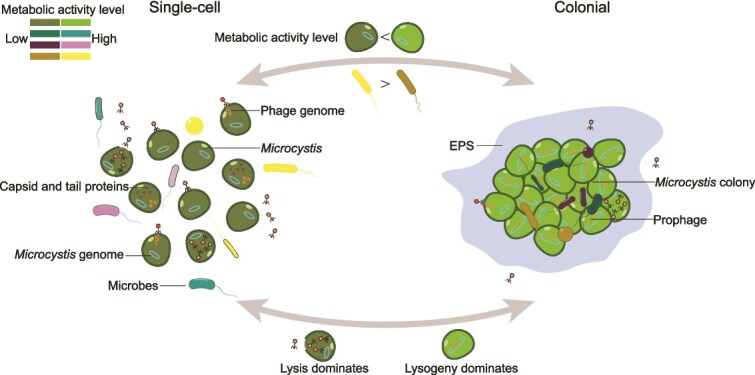
Metabolic activity and phage-host interactions in single-cell and colonial *Microcystis*. The left panel shows single-cell *Microcystis*, characterized by higher activity in associated microbes and lytic phage dominance. The right panel represents colonial *Microcystis*, where metabolic activity is concentrated in *Microcystis* and lysogeny dominates. Arrows indicate transitions between single-cell and colonial forms, driven by environmental factors and phage infection dynamics. Metabolic activity levels are represented using color brightness (Bright: high activity; Dark: low activity).

A final unique observation from this dataset is the difference between potential lysogens and lytically infected *Microcystis* cells in the colonial vs single cell samples. Studies on lysogen formation in *Microcystis* are in their infancy. But, the possibility of lysogen formation by Ma-LMM01-like phages was noted with the initial genomic sequencing of the virus [41], and seasonal field studies in *Taihu* demonstrated strong seasonal patterns in expression of genes to which lysogenic function is ascribed [18]*.* It should be noted that these markers were also detected in a transcriptome that was to be documenting the lysis of a population [42], although in that study 20% of the population persisted at the end of the experiment and these could have readily been lysogens. As with any bulk transcriptomic dataset, however, such patterns cannot resolve whether these transcripts originated from the same or different cells. The factors promoting or constraining these infection outcomes (and indeed even which partner makes that decision) remain unclear. In the present case, lysogen formation appears to be consistent with the hypothesis that this relationship forms/is selected for during life-at-high-density scenarios [43]: many lysogens demonstrate immunity to superinfection by similar viruses. Although existing evidence, including genomic observations and seasonal transcript patterns, strongly suggests that Ma-LMM01-like phages are capable of establishing lysogeny, our conclusions should still be interpreted in this context. During large scale *Microcystis* blooms, both the host and its virus can reach densities greater than 100 000 per ml [44]. At these densities viruses should contact potential hosts on a daily basis [19], effectively collapsing the bloom as is seen in other high density algal blooms [45, 46]. Given that colonies are a localized high density scenario, protection from superinfection would seem to be a necessary priority for members of the colony forming community. Going forward, it will be interesting to determine how colonies can mimic climax bloom communities as opposed to early season populations, which likely mimic single cells [38].

## Conclusion

Our study illuminates how colony formation restructures *Microcystis*’ metabolic activity and alters its interactions with both associated bacteria and phages (Fig. 4). Colonies appear to harbor a specialized microenvironment where *Microcystis* invests in sustaining a dense population, maintains putative fitness benefits such as enhanced stress tolerance, and supports an elevated propensity for lysogenic phage infection. By contrast, single-cell *Microcystis* undergoes more frequent lytic attacks, likely due to reduced cell density and diminished collective immunity. These findings highlight the dual ecological role of colony formation as both a protective refuge and a driver of complex host–virus dynamics.

Future controlled experiments—whereby environmental parameters, *Microcystis* colony density, and phage populations are systematically manipulated—will be essential to pinpoint the mechanistic underpinnings of lysogenic switching and to clarify the interplay between host competition, viral infection modes, and bloom persistence. Given the potentially key role of viruses in releasing toxins from the particulate to dissolved fraction in aquatic environments [47–49], understanding these processes are key to understanding system ecology and protecting our water resources. Cyanobacterial blooms and their associated toxins pose significant risks to aquatic ecosystems, drinking water supplies, and public health. By shedding light on these interactions, we can improve predictive modeling for bloom development and develop more effective strategies to mitigate their impacts, ultimately ensuring the availability of safe and sustainable water resources for ecosystems and human use alike.

## Supplementary Material

Supplementary_Information_2025_Oct_27_clean_wraf244

## Data Availability

Raw sequencing data for all 24 transcriptomic libraries are available at the NCBI Sequence Read Archive (SRA) under the accession number PRJNA1206705. The pangenomes and all scripts used in this study is available at https://github.com/cystis/microcystis_colony_phage, archived at Zenodo (DOI: 10.5281/zenodo.15780936).

## References

[1] Huisman J, Codd GA, Paerl HW et al. Cyanobacterial blooms. *Nat Rev Microbiol* 2018;16:471–83. 10.1038/s41579-018-0040-1 29946124

[2] Harke MJ, Steffen MM, Gobler CJ et al. A review of the global ecology, genomics, and biogeography of the toxic cyanobacterium. *Microcystis spp. Harmful Algae* 2016;54:4–20. 10.1016/j.hal.2015.12.007 28073480

[3] Wilhelm SW, Bullerjahn GS, McKay RML. The complicated and confusing ecology of *Microcystis* blooms. *MBio* 2020;11:e00529–0. 10.1128/mbio.00529-20 32605981 PMC7327167

[4] Yang G, Tang X, Wilhelm SW et al. Intermittent disturbance benefits colony size, biomass and dominance of *Microcystis* in Lake Taihu under field simulation condition. *Harmful Algae* 2020;99:101909. 10.1016/j.hal.2020.101909 33218435

[5] Huang X, Gu P, Wu H et al. Shift of calcium-induced *Microcystis aeruginosa* colony formation mechanism: from cell adhesion to cell division. *Environ Pollut* 2022;313:119997. 10.1016/j.envpol.2022.119997 35995295

[6] Xiao M, Li M, Reynolds CS. Colony formation in the cyanobacterium *Microcystis*. *Biol Rev* 2018;93:1399–420. 10.1111/brv.12401 29473286

[7] Yang Z, Kong F, Yang Z et al. Benefits and costs of the grazer-induced colony formation in *Microcystis aeruginosa*. *Ann Limnol Int J Limnol* 2009;45:203–8. 10.1051/limn/2009020

[8] Zhou Y, Cui X, Wu B et al. Microalgal extracellular polymeric substances (EPS) and their roles in cultivation, biomass harvesting, and bioproducts extraction. *Bioresour Technol* 2024;406:131054. 10.1016/j.biortech.2024.131054 38944317

[9] Le VV, Srivastava A, Ko S-R et al. *Microcystis* colony formation: extracellular polymeric substance, associated microorganisms, and its application. *Bioresour Technol* 2022;360:127610. 10.1016/j.biortech.2022.127610 35840029

[10] Feiner R, Argov T, Rabinovich L et al. A new perspective on lysogeny: prophages as active regulatory switches of bacteria. *Nat Rev Microbiol* 2015;13:641–50. 10.1038/nrmicro3527 26373372

[11] Menouni R, Hutinet G, Petit M-A et al. Bacterial genome remodeling through bacteriophage recombination. *FEMS Microbiol Lett* 2015;362:1–10. 10.1093/femsle/fnu022 25790500

[12] Hargreaves KR, Kropinski AM, Clokie MR. Bacteriophage behavioral ecology: how phages alter their bacterial host’s habits. *Bacteriophage* 2014;4:e29866. 10.4161/bact.29866 25105060 PMC4124054

[13] Howard-Varona C, Hargreaves KR, Abedon ST et al. Lysogeny in nature: mechanisms, impact and ecology of temperate phages. *ISME J* 2017;11:1511–20. 10.1038/ismej.2017.16 28291233 PMC5520141

[14] Davies EV, Winstanley C, Fothergill JL et al. The role of temperate bacteriophages in bacterial infection. *FEMS Microbiol Lett* 2016;363:fnw015. 10.1093/femsle/fnw015 26825679

[15] Duerkop BA, Clements CV, Rollins D et al. A composite bacteriophage alters colonization by an intestinal commensal bacterium. *Proc Natl Acad Sci* 2012;109:17621–6. 10.1073/pnas.1206136109 23045666 PMC3491505

[16] Paul JH . Prophages in marine bacteria: dangerous molecular time bombs or the key to survival in the seas? *ISME J* 2008;2:579–89. 10.1038/ismej.2008.35 18521076

[17] Touchon M, Moura De Sousa JA, Rocha EP. Embracing the enemy: the diversification of microbial gene repertoires by phage-mediated horizontal gene transfer. *Curr Opin Microbiol* 2017;38:66–73. 10.1016/j.mib.2017.04.010 28527384

[18] Stough JMA, Tang X, Krausfeldt LE et al. Molecular prediction of lytic *vs* lysogenic states for *Microcystis* phage: metatranscriptomic evidence of lysogeny during large bloom events. *PLoS One* 2017;12:e0184146. 10.1371/journal.pone.0184146 28873456 PMC5584929

[19] Murray AG, Jackson GA. Viral dynamics: a model of the effects of size, shape, motion and abundance of single-celled planktonic organisms and other particles. *Mar Ecol Prog Ser* 1992;89:103–16.

[20] Pound HL, Gann ER, Wilhelm SW. A comparative study of metatranscriptomic assessment methods to characterize blooms. *Limnol Oceanogr Methods* 2021;19:846–54. 10.1002/lom3.10465 35528780 PMC9075346

[21] Fu L, Niu B, Zhu Z et al. CD-HIT: accelerated for clustering the next-generation sequencing data. *Bioinformatics* 2012;28:3150–2. 10.1093/bioinformatics/bts565 23060610 PMC3516142

[22] Tatusova T, DiCuccio M, Badretdin A et al. NCBI prokaryotic genome annotation pipeline. *Nucleic Acids Res* 2016;44:6614–24. 10.1093/nar/gkw569 27342282 PMC5001611

[23] Cantalapiedra CP, Hernández-Plaza A, Letunic I et al. eggNOG-mapper v2: functional annotation, orthology assignments, and domain prediction at the metagenomic scale. *Mol Biol Evol* 2021;38:5825–9. 10.1093/molbev/msab293 34597405 PMC8662613

[24] Chen S, Zhou Y, Chen Y et al. Fastp: an ultra-fast all-in-one FASTQ preprocessor. *Bioinformatics* 2018;34:i884–90. 10.1093/bioinformatics/bty560 30423086 PMC6129281

[25] Bushnell B . BBMap: A Fast, Accurate, Splice-Aware Aligner. Berkeley, CA: Lawrence Berkeley National Laboratory; 2014.

[26] Li D, Luo R, Liu C-M et al. MEGAHIT v1.0: a fast and scalable metagenome assembler driven by advanced methodologies and community practices. *Methods* 2016;102:3–11. 10.1016/j.ymeth.2016.02.020 27012178

[27] Gurevich A, Saveliev V, Vyahhi N et al. QUAST: quality assessment tool for genome assemblies. *Bioinformatics* 2013;29:1072–5. 10.1093/bioinformatics/btt086 23422339 PMC3624806

[28] Liao Y, Smyth GK, Shi W. featureCounts: an efficient general purpose program for assigning sequence reads to genomic features. *Bioinformatics* 2014;30:923–30. 10.1093/bioinformatics/btt656 24227677

[29] Zhu W, Lomsadze A, Borodovsky M. *Ab initio* gene identification in metagenomic sequences. *Nucleic Acids Res* 2010;38:e132–2. 10.1093/nar/gkq275 20403810 PMC2896542

[30] Wood DE, Lu J, Langmead B. Improved metagenomic analysis with kraken 2. *Genome Biol* 2019;20:257. 10.1186/s13059-019-1891-0 31779668 PMC6883579

[31] R Core Team . R: a language and environment for statistical computing. Vienna, Austria. *R Foundation for Statistical Computing* 2024. https://www.r-project.org/.

[32] Xu S, Hu E, Cai Y et al. Using clusterProfiler to characterize multiomics data. *Nat Protoc* 2024;19:3292–320. 10.1038/s41596-024-01020-z 39019974

[33] Wickham H . ggplot2: Elegant Graphics for Data Analysis. New York: Springer-Verlag, 2016, https://ggplot2.tidyverse.org/.

[34] Love MI, Huber W, Anders S. Moderated estimation of fold change and dispersion for RNA-seq data with DESeq2. *Genome Biol* 2014;15:550. 10.1186/s13059-014-0550-8 25516281 PMC4302049

[35] Yang F, Jin H, Wang X-Q et al. Genomic analysis of Mic1 reveals a novel freshwater long-tailed cyanophage. *Front Microbiol* 2020;11:484. 10.3389/fmicb.2020.00484 32322241 PMC7156551

[36] Zepernick BN, Niknejad DJ, Stark GF et al. Morphological, physiological, and transcriptional responses of the freshwater diatom *Fragilaria crotonensis* to elevated pH conditions. *Front Microbiol* 2022;13:1044464. 10.3389/fmicb.2022.1044464 36504786 PMC9732472

[37] Gao N, Xia M, Dai J et al. Both widespread PEP-CTERM proteins and exopolysaccharides are required for floc formation of *Zoogloea resiniphila* and other activated sludge bacteria. *Environ Microbiol* 2018;20:1677–92. 10.1111/1462-2920.14080 29473278

[38] Tang X, Krausfeldt LE, Shao K et al. Seasonal gene expression and the ecophysiological implications of toxic *Microcystis aeruginosa* blooms in Lake Taihu. *Environ Sci Technol* 2018;52:11049–59. 10.1021/acs.est.8b01066 30168717

[39] Morris JJ, Lenski RE, Zinser ER. The black queen hypothesis: evolution of dependencies through adaptive gene loss. *mBio* 2012;3:e00036–12. 10.1128/mbio.00036-12 22448042 PMC3315703

[40] Pound HL, Martin RM, Sheik CS et al. Environmental studies of cyanobacterial harmful algal blooms should include interactions with the dynamic microbiome. *Environ Sci Technol* 2021;55:12776–9. 10.1021/acs.est.1c04207 34529413 PMC9017748

[41] Yoshida T, Nagasaki K, Takashima Y et al. Ma-LMM01 infecting toxic *Microcystis aeruginosa* illuminates diverse cyanophage genome strategies. *J Bacteriol* 2008;190:1762–72. 10.1128/JB.01534-07 18065537 PMC2258655

[42] Morimoto D, Kimura S, Sako Y et al. Transcriptome analysis of a bloom-forming cyanobacterium *Microcystis aeruginosa* during Ma-LMM01 phage infection. *Front Microbiol* 2018;9:2. 10.3389/fmicb.2018.00002 29403457 PMC5780444

[43] Knowles B, Silveira CB, Bailey BA et al. Lytic to temperate switching of viral communities. *Nature* 2016;531:466–70. 10.1038/nature17193 26982729

[44] Rozon RM, Short SM. Complex seasonality observed amongst diverse phytoplankton viruses in the bay of Quinte, an embayment of Lake Ontario. *Freshw Biol* 2013;58:2648–63. 10.1111/fwb.12241

[45] Gann ER, Truchon AR, Papoulis SE et al. *Aureococcus anophagefferens* (Pelagophyceae) genomes improve evaluation of nutrient acquisition strategies involved in brown tide dynamics. *J Phycol* 2022;58:146–60. 10.1111/jpy.13221 34773248

[46] McKindles KM, Manes MA, DeMarco JR et al. Dissolved microcystin release coincident with lysis of a bloom dominated by *Microcystis* spp. in western Lake Erie attributed to a novel cyanophage. *Appl Environ Microbiol* 2020;86:e01397–20. 10.1128/AEM.01397-20 32859600 PMC7642080

[47] Steffen MM, Davis TW, McKay RML et al. Ecophysiological examination of the Lake Erie *Microcystis* bloom in 2014: linkages between biology and the water supply shutdown of Toledo. *OH Environ Sci Technol* 2017;51:6745–55. 10.1021/acs.est.7b00856 28535339

[48] Steffen MM, Belisle BS, Watson SB et al. Metatranscriptomic evidence for co-occurring top-down and bottom-up controls on toxic cyanobacterial communities. *Appl Environ Microbiol* 2015;81:3268–76. 10.1128/AEM.04101-14 25662977 PMC4393433

[49] Lee V, Meza-Padilla I, Nissimov JI. Virus infection of a freshwater cyanobacterium contributes significantly to the release of toxins through cell lysis. *Microorganisms* 2025;13:486. 10.3390/microorganisms13030486 40142379 PMC11944701

