# Correction to: Contrasting viral infection strategies for single cell and colonial *Microcystis* populations consistent with Black Queen dynamics

**DOI:** 10.1093/ismejo/wrag117

**Published:** 2026-06-05

This is a correction to: Xuhui Huang, Emily E Chase, Brittany N Zepernick, Robbie M Martin, Lauren E Krausfeldt, Helena L Pound, Hanqi Wu, Zheng Zheng, Steven W Wilhelm, Contrasting viral infection strategies for single cell and colonial *Microcystis* populations consistent with Black Queen dynamics, *The ISME Journal*, Volume 19, Issue 1, January 2025, wraf244, https://doi.org/10.1093/ismejo/wraf244

In the originally published version of this manuscript, an earlier version of Figure 3 was used. The correct Figure 3 is as follows:


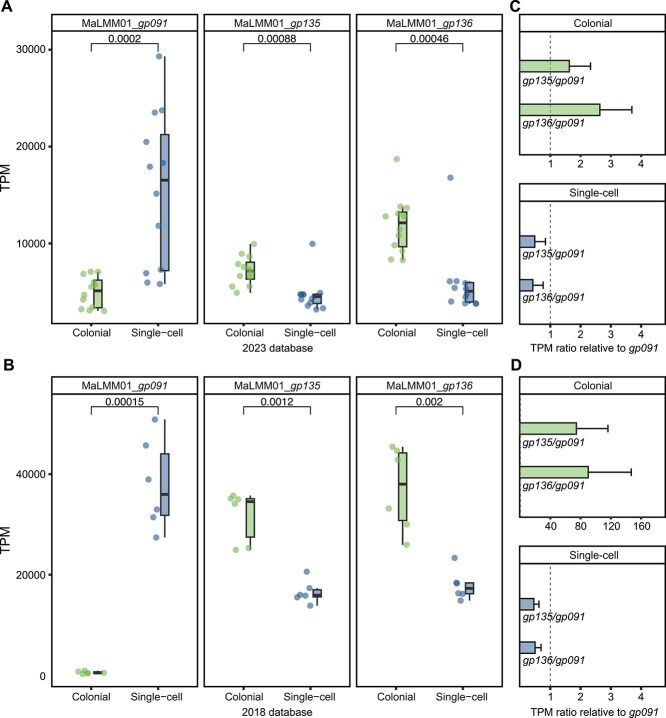


These details have been corrected only in this correction notice to preserve the published version of record.

