# Supplementary material for: Contrasting viral infection strategies for single cell and colonial Microcystis populations consistent with Black Queen dynamics
Source: ISME J. 2025 Nov 3;19(1):wraf244. doi: 10.1093/ismejo/wraf244 (PMC12642866; doi:10.1093/ismejo/wraf244)
Supplement: Supplementary_Information_2025_Oct_27_clean_wraf244 [file supplementary_information_2025_oct_27_clean_wraf244.pdf]

Supplementary Information for:

Contrasting viral infection strategies for single cell and colonial *Microcystis* populations consistent with Black Queen dynamics

X. Huang[1,2], EE Chase[2], BN Zepernick[2], RM Martin[2], LE Krausfeldt[2], HL Pound[2], H. Wu[1], Z. Zheng[*1] and SW Wilhelm[*2]

1. Department of Environmental Science and Engineering, Fudan University, Shanghai, PR China

2. Department of Microbiology, The University of Tennessee, Knoxville TN

Address for contact information: wilhelm@utk.edu, zzhenghj@fudan.edu.cn

METHODS

Environmental Parameters of 2023 samples

Water temperature was measured in situ using a multiparameter water quality sonde (Hach DS5). Total nitrogen (TN) and total phosphorus (TP) were analyzed following filtration through a 0.45 μm water system filter membrane. TN was determined using a TOC analyzer (Shimadzu), and TP was quantified using the molybdenum blue colorimetric method.

Sample collection of 2018 samples

Surface water samples were collected from a dock that stretches approximately 100 yards into Meilang Bay near the TLLER field station on *Taihu* on 08/18/2018 at 13:00 and 08/19/2018 at 7:00. To enrich for colonies, water was filtered through a 28 μm mesh while the < 28 μm filtrate was captured as the representative single-cell community. Duplicate samples were collected at each time point and both fractions were filtered through Sterivex, preserved with RNA*later* and stored at -80 until processing.

RNA extraction and sequencing of 2018 samples

Total RNA was extracted using a previously described phenol–chloroform method followed by ethanol precipitation[1]. Genomic DNA was removed using the Turbo DNA-free kit (Ambion, Austin, TX, USA). RNA integrity and concentration were assessed with the Qubit RNA HS Assay Kit (Invitrogen, Waltham, MA, USA). Samples exhibiting residual DNA, as indicated by Qubit quantification, underwent an additional DNase treatment. Purified RNA was subsequently subjected to library preparation, rRNA depletion, and sequencing on the NextSeq platform (Illumina) using the Epidemiology Ribo-Zero Gold rRNA Removal Kit.

Supplementary Table 1: Information of each sampling site. The first letter of each sample name indicates the morphology of the sample, with C representing colony and S representing single cell. The second segment represents the sampling site (e.g., S2), and the last number indicates the replicate number.

Sampling site	Date	Time	Longitude	Latitude	Colonial samples	Single-cell samples	Water temperature (°C)	TN (mg/L)	TP (mg/L)
S1	8/26/2023	10:02	120°01'21''E	31°24'48''N	C_S1_1<br>C_S1_2<br>C_S1_3	S_S1_1<br>S_S1_2<br>S_S1_3	30.2	2.04	0.23
S2	8/26/2023	10:13	120°00'10''E	31°23'28''N	C_S2_1<br>C_S2_2<br>C_S2_3	S_S2_1<br>S_S2_2<br>S_S2_3	29.8	2.48	0.37
S3	8/26/2023	10:27	119°58'30''E	31°20'55''N	C_S3_1<br>C_S3_2<br>C_S3_3	S_S3_1<br>S_S3_2<br>S_S3_3	29.9	5.12	1.11
S4	8/26/2023	11:15	120°02'43''E	31°23'51''N	C_S4_1<br>C_S4_2<br>C_S4_3	S_S4_1<br>S_S4_2<br>S_S4_3	28.8	3.51	0.27

Supplementary Table 2: Summary of 16 complete *Microcystis* genomes used for the construction of the *Microcystis* pangenome.

Organism Scientific Name	Organism Qualifier	Taxonomy id	Assembly Accession	Source	Size	Gene Count
Microcystis aeruginosa NIES-843	strain: NIES-843	449447	GCF_000010625.1	RefSeq	5842795	5792
Microcystis aeruginosa NIES-2549	strain: NIES-2549	1641812	GCF_000981785.2	RefSeq	4301200	4087
Microcystis panniformis FACHB-1757	strain: FACHB-1757	1638788	GCF_001264245.1	RefSeq	5686839	5640
Microcystis aeruginosa NIES-2481	strain: NIES-2481	1698524	GCF_001704955.2	RefSeq	4440545	4242
Microcystis aeruginosa PCC 7806SL	strain: PCC 7806SL	1903187	GCF_002095975.1	RefSeq	5139339	4936
Microcystis sp. MC19	strain: MC19	1967666	GCF_003019735.1	RefSeq	5020243	4934
Microcystis viridis NIES-102	strain: NIES-102	213615	GCF_003945305.1	RefSeq	5874197	5786
Microcystis aeruginosa FD4	strain: FD4	2686288	GCF_009792235.1	RefSeq	5493112	5530
Microcystis aeruginosa NIES-298	strain: NIES-298	449468	GCF_010196425.1	RefSeq	5015081	4823
Microcystis aeruginosa	strain: NIES-88	1126	GCF_019704275.1	RefSeq	5501105	5588
Microcystis aeruginosa FACHB-905 = DIANCHI905	strain: DIANCHI905	267865	GCF_021172085.1	RefSeq	5103104	4909
Microcystis aeruginosa str. Chao 1910	strain: Chao 1910	2945101	GCF_026222535.1	RefSeq	5669822	5720
Microcystis aeruginosa PCC 7806	strain: PCC 7806	267872	GCF_030553035.1	RefSeq	5103923	4908
Microcystis aeruginosa NRERC-214	strain: NRERC-214	2528657	GCF_031754835.1	RefSeq	4981678	·4671
Microcystis aeruginosa 1339	strain: 1339	3113711	GCF_038396575.1	RefSeq	4749293	4559
Microcystis aeruginosa PCC 7806	strain: PCC 7806	267872	GCF_041506625.1	RefSeq	5096229	4894

Supplementary Table 3: Summary of 41 complete *Microcystis* phage genomes used for the construction of the *Microcystis* phage pangenome.

Phage Name	Genome Size & Type	Accession	GI Number
Microcystis phage Ma-LMM01 DNA, complete genome	162,109 bp circular DNA	NC_008562.1	117530171
Microcystis phage MaMV-DC, complete genome	169,223 bp circular DNA	NC_029002.1	971764724
Microcystis phage vB_MaeS-yong1, complete genome	43,665 bp linear DNA	MT855965.1	1896828356
Microcystis phage Mae-JY35, complete genome	42,400 bp linear DNA	PP438426.1	2725671406
Microcystis phage Mel-JY34, complete genome	44,433 bp linear DNA	PP438425.1	2725671342
Microcystis phage Mel-JY33, complete genome	47,173 bp linear DNA	PP438424.1	2725671287
Microcystis phage Mwe-JY31, complete genome	22,207 bp linear DNA	PP438423.1	2725671268
Microcystis phage Mae-JY30, complete genome	61,694 bp linear DNA	PP438422.1	2725671179
Microcystis phage Mae-JY29, complete genome	37,236 bp linear DNA	PP438421.1	2725671124
Microcystis phage Mae-JY28, complete genome	40,271 bp linear DNA	PP438420.1	2725671064
Microcystis phage Mwe-JY26, complete genome	101,250 bp linear DNA	PP438418.1	2725670807
Microcystis phage Mwe-JY25, complete genome	41,316 bp linear DNA	PP438417.1	2725670744
Microcystis phage Mae-JY24, complete genome	43,641 bp linear DNA	PP438416.1	2725670670
Microcystis phage Mae-JY22, complete genome	41,362 bp linear DNA	PP438414.1	2725670512
Microcystis phage Mvi-JY20, complete genome	107,975 bp linear DNA	PP438412.1	2725670303
Microcystis phage Mwe-JY13, complete genome	41,296 bp linear DNA	PP438406.1	2725669990
Microcystis phage Me-ZS1, complete genome	49,665 bp linear DNA	MK069556.2	1576867779
Microcystis phage Mel-Yong916-1, complete genome	171,051 bp circular DNA	OQ560327.1	2496141310
Microcystis phage MaMV-DL02, complete genome	177,212 bp linear DNA	PP681324.1	2866157454
Microcystis phage MaMV-DL01, complete genome	177,213 bp linear DNA	PP681323.1	2866157240
Microcystis phage MaMV-CH02, complete genome	172,789 bp linear DNA	PP681322.1	2866157033
Microcystis phage MaMV-CH01, complete genome	171,437 bp linear DNA	PP681321.1	2866156824
Microcystis phage Mae-JY09, complete genome	38,976 bp linear DNA	PP395641.1	2693695438
Microcystis phage Mwe-JY08, complete genome	45,453 bp linear DNA	PP395640.1	2693695369
Microcystis phage Mwe-JY07, complete genome	58,068 bp linear DNA	PP395639.1	2693695301
Microcystis phage Mwe-JY05, complete genome	71,137 bp linear DNA	PP395638.1	2693695201
Microcystis phage Mae-JY04, complete genome	40,260 bp linear DNA	PP395637.1	2693695129
Microcystis phage Mel-JY03, complete genome	40,253 bp linear DNA	PP395636.1	2693695005
Microcystis phage Mae-JY02, complete genome	37,464 bp linear DNA	PP395635.1	2693694904
Microcystis phage Mel-JY01, complete genome	190,001 bp linear DNA	PP395634.1	2693694637
Microcystis phage MaAM05, complete genome	273,876 bp linear DNA	MW495066.1	1990399386
Microcystis phage MJing1, complete genome	42,068 bp linear DNA	OQ540923.1	2552392052
Microcystis phage Mae-Yong924-2, complete genome	40,438 bp linear DNA	MZ570427.1	2074143851
Microcystis phage Mae-Yong1326-1, complete genome	48,822 bp linear DNA	OP028995.1	2282035451
Microcystis phage vB_MweS-yong2, complete genome	44,530 bp linear DNA	OM681334.1	2209435578
Microcystis phage MinS1, complete genome	49,966 bp circular DNA	MZ923504.1	2177696787
Microcystis phage Mea-Yong924-1, complete genome	40,325 bp circular DNA	MZ447863.1	2067455506
Microcystis phage Mwe-Yong1112-1, complete genome	39,679 bp linear DNA	MZ436628.1	2067455452
Microcystis phage MaeS, complete genome	79,995 bp linear DNA	MT362618.1	1841757278
Microcystis phage MACPNOA1, complete genome	42,473 bp linear DNA	KY697807.1	1168017638
Microcystis phage Mic1, complete genome	92,627 bp circular DNA	MN013189.1	1682781200

Supplementary Table 4: Summary of sequence information for Lake Taihu metatranscriptomic libraries collected on August 26, 2023. The first letter of each sample name indicates the morphology of the sample, with C representing colony and S representing single cell. The second segment represents the sampling site (e.g., S2), and the last number indicates the replicate number.

Sample	Raw reads count	Total bases of raw reads	Trimmed reads count	Trimmed reads percentage (%)	mRNA reads count	mRNA reads percentage (%)	Reads mapped to the *Microcystis* pangenome count	*Microcystis* pangenome -mapped reads percentage (%)	Reads mapped to the co-assembly count	Co-assembly-mapped reads percentage (%)
**C_S1_1**	1.55E+08	2.33E+10	1.52E+08	98.17%	1.52E+08	99.93%	5.53E+07	36.49%	1.34E+08	88.29%
**C_S1_2**	1.58E+08	2.39E+10	1.54E+08	97.50%	1.54E+08	99.92%	6.85E+07	44.43%	1.4E+08	90.65%
**C_S1_3**	9.74E+07	1.47E+10	9.53E+07	97.86%	9.52E+07	99.95%	3.32E+07	34.9%	8.39E+07	88.11%
**C_S2_1**	1.4E+08	2.12E+10	1.37E+08	97.95%	1.37E+08	99.75%	5.13E+07	37.41%	1.23E+08	90.03%
**C_S2_2**	1.18E+08	1.78E+10	1.15E+08	97.57%	1.15E+08	99.71%	4.37E+07	38.1%	1.03E+08	89.79%
**C_S2_3**	8.99E+07	1.36E+10	8.78E+07	97.69%	8.78E+07	99.96%	2.79E+07	31.79%	7.83E+07	89.16%
**C_S3_1**	1.4E+08	2.11E+10	1.32E+08	94.90%	1.31E+08	99.05%	5.95E+07	45.38%	1.21E+08	92.48%
**C_S3_2**	1.67E+08	2.52E+10	1.63E+08	97.77%	1.63E+08	99.84%	7.31E+07	44.89%	1.5E+08	91.97%
**C_S3_3**	9.68E+07	1.46E+10	9.58E+07	98.98%	9.43E+07	98.35%	4.3E+07	45.6%	8.61E+07	91.31%
**C_S4_1**	1.35E+08	2.04E+10	1.28E+08	94.87%	1.28E+08	99.94%	5.61E+07	43.8%	1.17E+08	91.09%
**C_S4_2**	1.08E+08	1.62E+10	1.06E+08	98.37%	1.06E+08	99.90%	4.17E+07	39.45%	9.66E+07	91.36%
**C_S4_3**	9.6E+07	1.45E+10	9.36E+07	97.44%	9.35E+07	99.95%	3.42E+07	36.54%	8.3E+07	88.75%
**S_S1_1**	1.15E+08	1.74E+10	1.12E+08	97.78%	1.12E+08	99.94%	3.59E+07	31.95%	9.83E+07	87.52%
**S_S1_2**	1.14E+08	1.73E+10	1.11E+08	97.19%	1.11E+08	99.92%	2.21E+07	19.93%	8.98E+07	80.82%
**S_S1_3**	1.14E+08	1.72E+10	1.1E+08	96.68%	1.1E+08	99.89%	2.96E+07	26.86%	9.32E+07	84.52%
**S_S2_1**	1.13E+08	1.7E+10	1.1E+08	97.07%	1.09E+08	99.87%	1.62E+07	14.84%	8.46E+07	77.29%
**S_S2_2**	1.08E+08	1.63E+10	1.06E+08	98.21%	1.06E+08	99.93%	1.27E+07	11.99%	8.49E+07	80.36%
**S_S2_3**	1.02E+08	1.54E+10	9.99E+07	98.08%	9.97E+07	99.84%	1.72E+07	17.25%	8.53E+07	85.53%
**S_S3_1**	1.37E+08	2.07E+10	1.34E+08	97.43%	1.34E+08	99.95%	2.7E+07	20.19%	1.18E+08	88.27%
**S_S3_2**	1.46E+08	2.21E+10	1.43E+08	97.79%	1.43E+08	99.93%	3.35E+07	23.41%	1.28E+08	89.4%
**S_S3_3**	1.36E+08	2.06E+10	1.33E+08	97.52%	1.33E+08	99.93%	3.5E+07	26.36%	1.19E+08	89.36%
**S_S4_1**	1.26E+08	1.9E+10	1.22E+08	96.46%	1.21E+08	99.87%	1.48E+07	12.19%	9.7E+07	79.93%
**S_S4_2**	1.38E+08	2.08E+10	1.33E+08	96.26%	1.32E+08	99.83%	1.73E+07	13.03%	1.08E+08	81.65%
**S_S4_3**	1.02E+08	1.54E+10	9.95E+07	97.68%	9.94E+07	99.91%	1.07E+07	10.73%	8.02E+07	80.61%

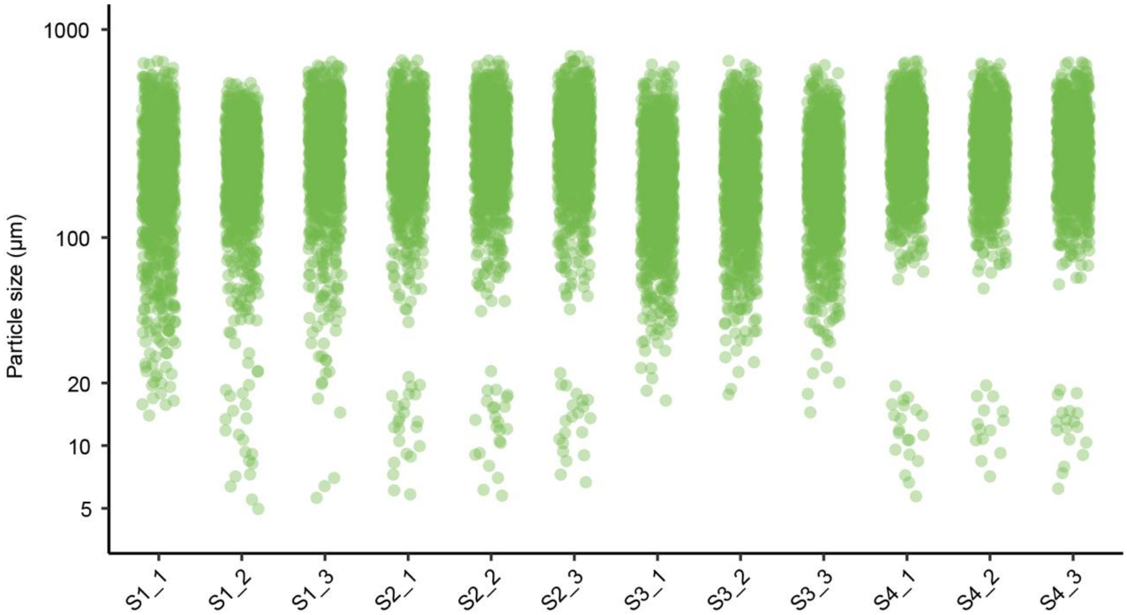

Supplementary Figure 1: Size distribution of 12 colonial samples collected in 2023. To visualize size distribution, each particle size range was assigned a number of points proportional to its volumetric percentage, scaled to a total of 1000 points per sample.

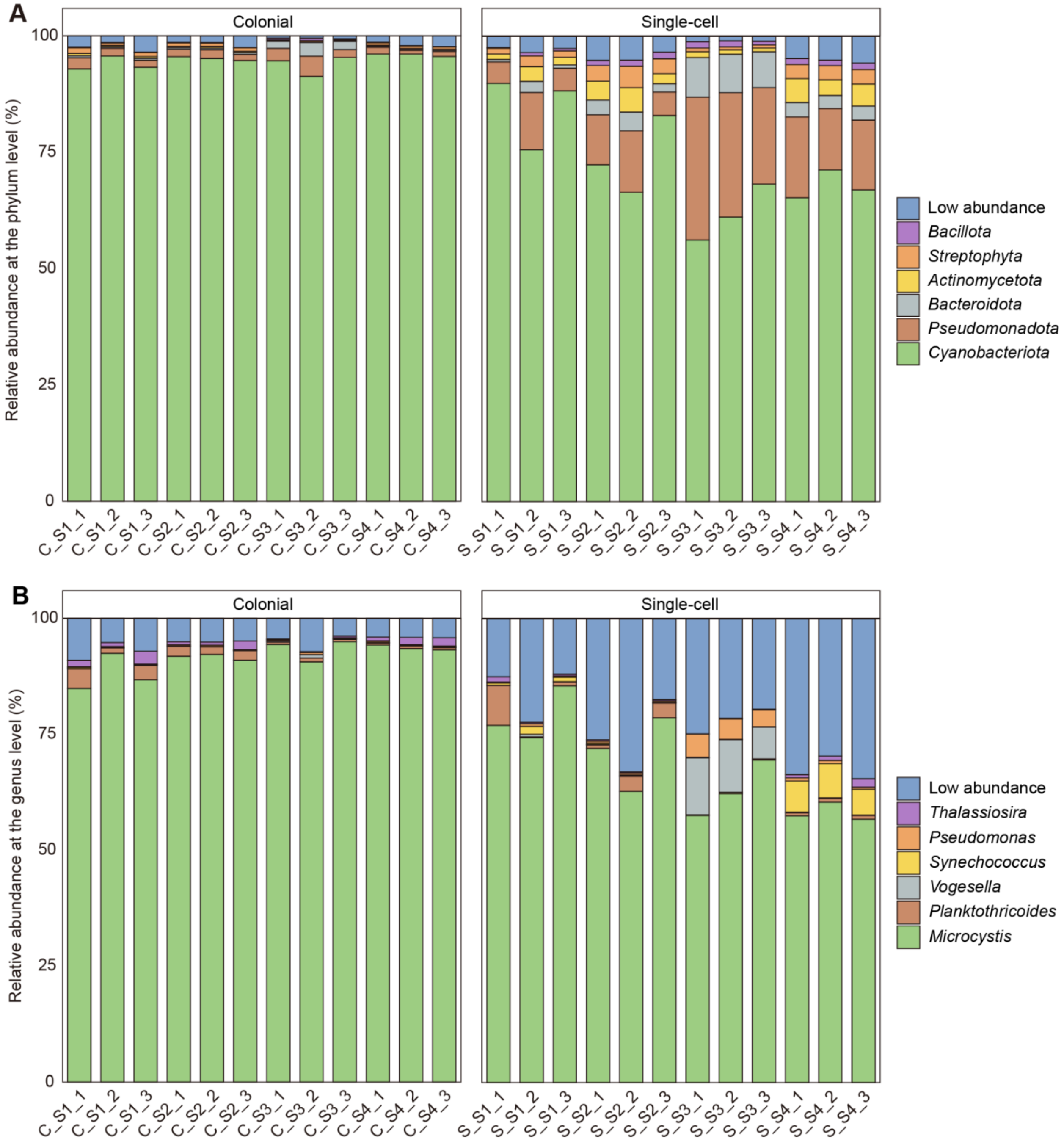

Supplementary Figure 2: Phylum- (**A**) and genus-level (**B**) distribution of colonial and single-cell samples in the 2023 database. Each bar represents a single sample, with the relative abundance of taxa shown as a percentage. The number of samples is as follows: colonial (n = 12) and single-cell (n = 12).

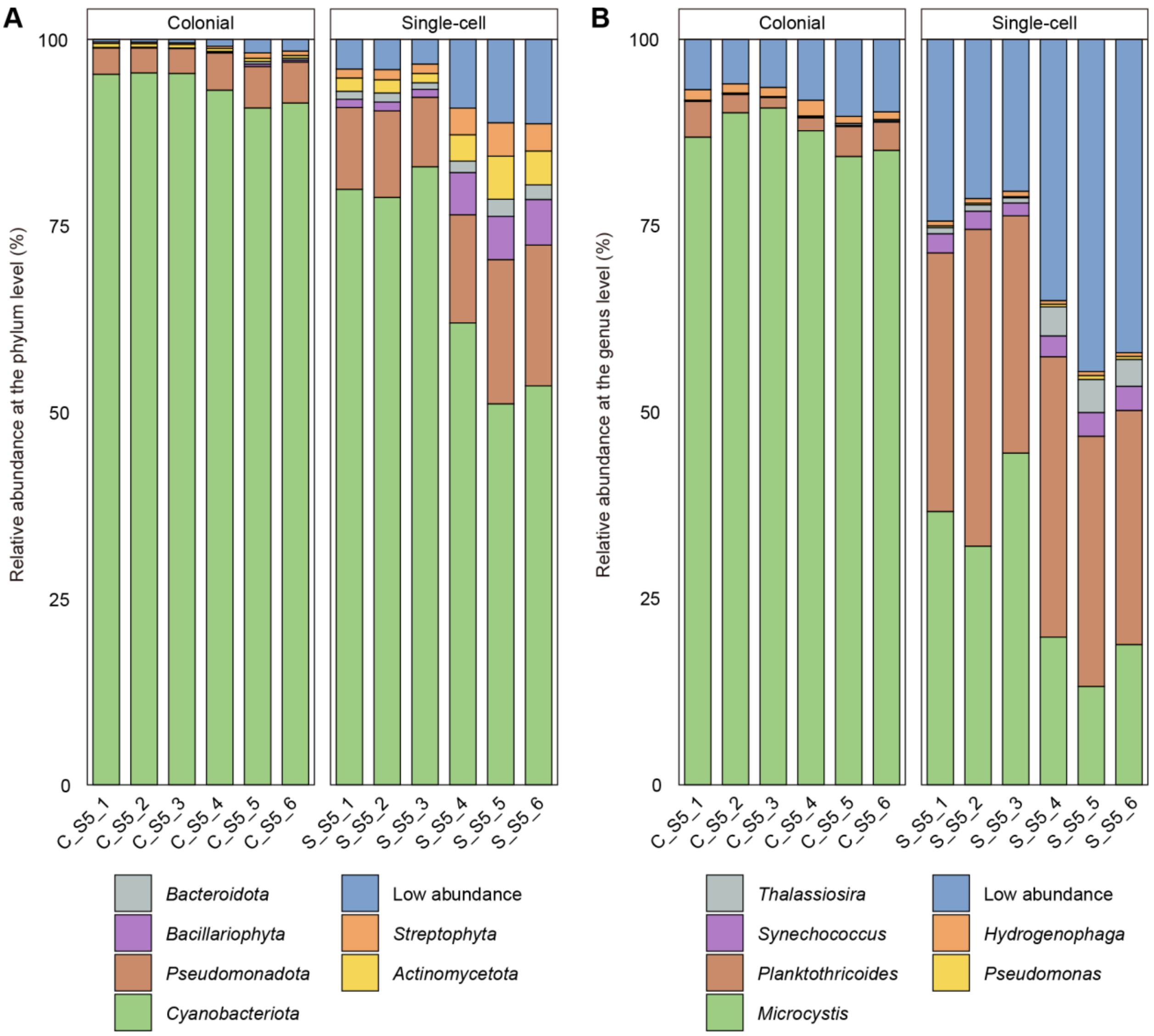

Supplementary Figure 3: Phylum- (**A**) and genus-level (**B**) distribution of colonial and single-cell samples in the 2018 database. Each bar represents a single sample, with the relative abundance of taxa shown as a percentage. The number of samples is as follows: colonial (n = 6) and single-cell (n = 6).

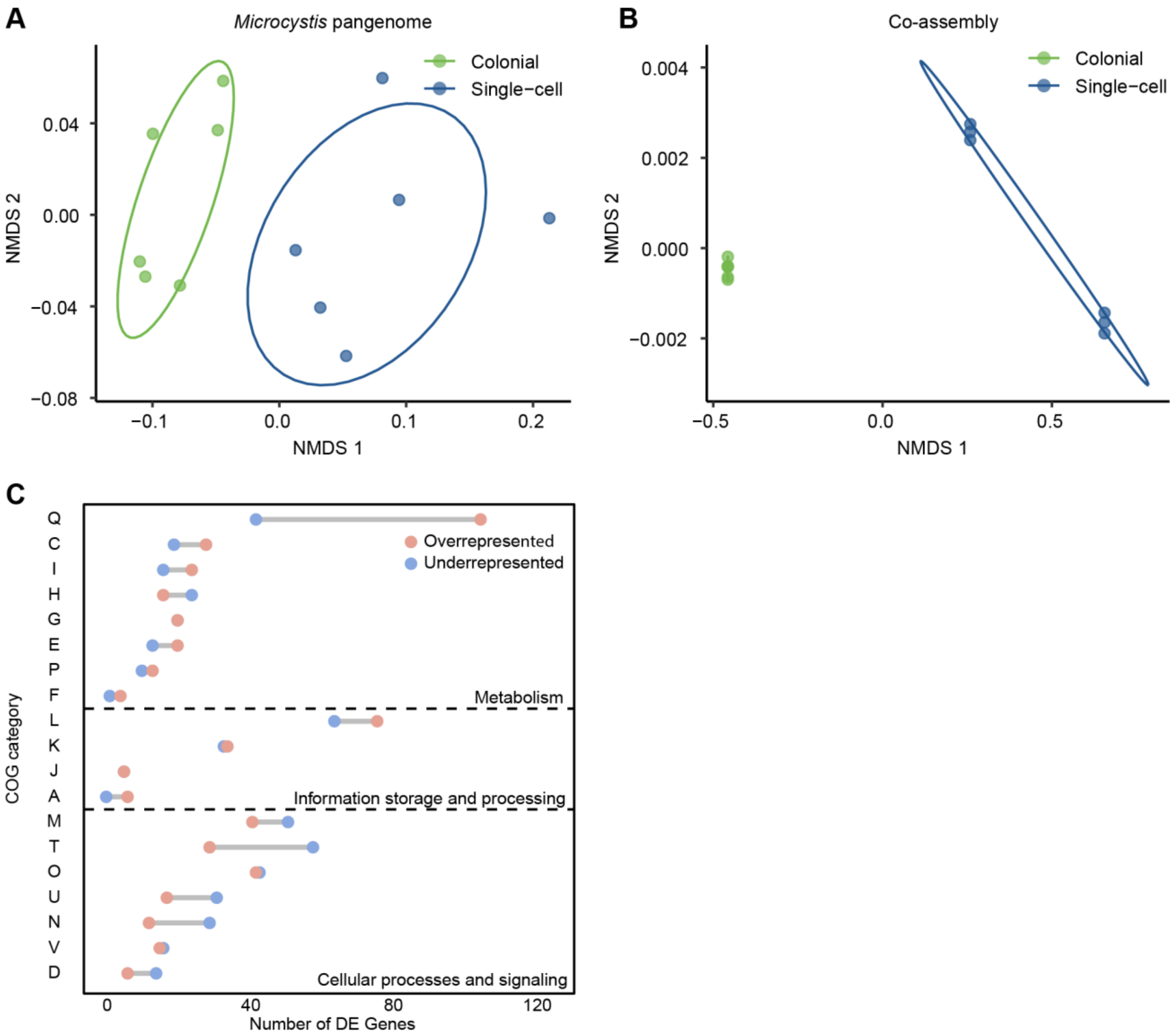

Supplementary Figure 4: Functional distribution based on the co-assembly and *Microcystis* pangenome. **A**, **B**, Nonmetric multidimensional scaling (NMDS) plot based on Bray–Curtis dissimilarity of transcriptomic data from 2018 samples, mapped to the co-assembly (**A**) and the *Microcystis* pangenome (**B**), using transcript per million (TPM) normalized data. Ellipses cover 68% of the data for each form. **C**, Distribution of differentially expressed (DE) *Microcystis* genes across COG functional categories in the 2018 dataset.

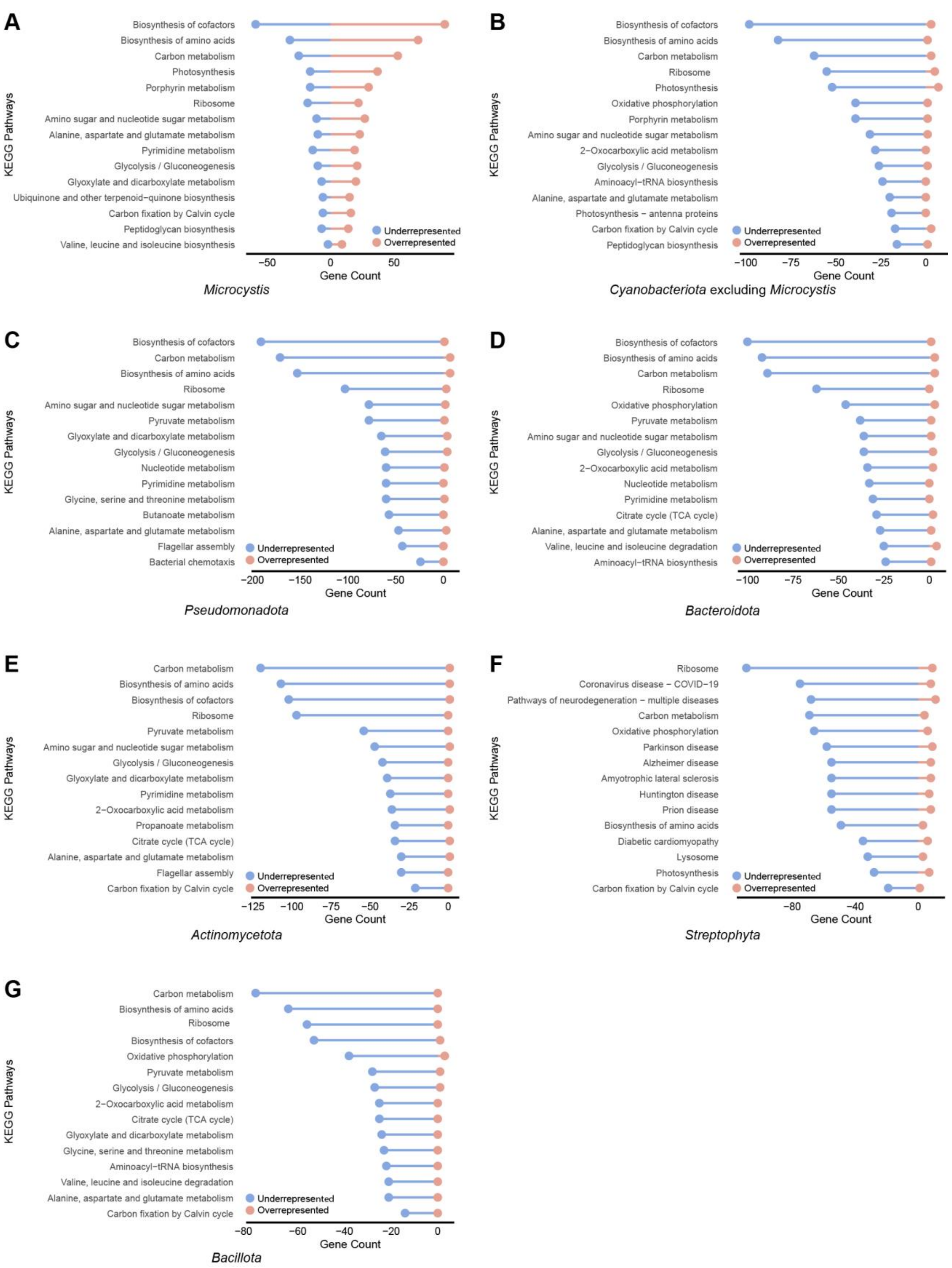
A
Biosynthesis of cofactors
Biosynthesis of amino acids
Carbon metabolism
Photosynthesis
Porphyrin metabolism
Ribosome
Amino sugar and nucleotide sugar metabolism
Alanine, aspartate and glutamate metabolism
Pyrimidine metabolism
Glycolysis / Gluconeogenesis
Glyoxylate and dicarboxylate metabolism
Ubiquinone and other terpenoid−quinone biosynthesis
Carbon fixation by Calvin cycle
Peptidoglycan biosynthesis
Valine, leucine and isoleucine biosynthesis
KEGG Pathways
Underrepresented
Overrepresented
−50
0
50
Gene Count
Microcystis
B
Biosynthesis of cofactors
Biosynthesis of amino acids
Carbon metabolism
Ribosome
Photosynthesis
Oxidative phosphorylation
Porphyrin metabolism
Amino sugar and nucleotide sugar metabolism
2−Oxocarboxylic acid metabolism
Glycolysis / Gluconeogenesis
Aminoacyl−tRNA biosynthesis
Alanine, aspartate and glutamate metabolism
Photosynthesis − antenna proteins
Carbon fixation by Calvin cycle
Peptidoglycan biosynthesis
KEGG Pathways
Underrepresented
Overrepresented
−100
−75
−50
−25
0
Gene Count
Cyanobacteriota excluding Microcystis
C
Biosynthesis of cofactors
Carbon metabolism
Biosynthesis of amino acids
Ribosome
Amino sugar and nucleotide sugar metabolism
Pyruvate metabolism
Glyoxylate and dicarboxylate metabolism
Glycolysis / Gluconeogenesis
Nucleotide metabolism
Pyrimidine metabolism
Glycine, serine and threonine metabolism
Butanoate metabolism
Alanine, aspartate and glutamate metabolism
Flagellar assembly
Bacterial chemotaxis
KEGG Pathways
Underrepresented
Overrepresented
−200
−150
−100
−50
0
Gene Count
Pseudomonadota
D
Biosynthesis of cofactors
Biosynthesis of amino acids
Carbon metabolism
Ribosome
Oxidative phosphorylation
Pyruvate metabolism
Amino sugar and nucleotide sugar metabolism
Glycolysis / Gluconeogenesis
2−Oxocarboxylic acid metabolism
Nucleotide metabolism
Pyrimidine metabolism
Citrate cycle (TCA cycle)
Alanine, aspartate and glutamate metabolism
Valine, leucine and isoleucine degradation
Aminoacyl−tRNA biosynthesis
KEGG Pathways
Underrepresented
Overrepresented
−100
−75
−50
−25
0
Gene Count
Bacteroidota
E
Carbon metabolism
Biosynthesis of amino acids
Biosynthesis of cofactors
Ribosome
Pyruvate metabolism
Amino sugar and nucleotide sugar metabolism
Glycolysis / Gluconeogenesis
Glyoxylate and dicarboxylate metabolism
Pyrimidine metabolism
2−Oxocarboxylic acid metabolism
Propanoate metabolism
Citrate cycle (TCA cycle)
Alanine, aspartate and glutamate metabolism
Flagellar assembly
Carbon fixation by Calvin cycle
KEGG Pathways
Underrepresented
Overrepresented
−125
−100
−75
−50
−25
0
Gene Count
Actinomycetota
F
Ribosome
Coronavirus disease − COVID−19
Pathways of neurodegeneration − multiple diseases
Carbon metabolism
Oxidative phosphorylation
Parkinson disease
Alzheimer disease
Amyotrophic lateral sclerosis
Huntington disease
Prion disease
Biosynthesis of amino acids
Diabetic cardiomyopathy
Lysosome
Photosynthesis
Carbon fixation by Calvin cycle
KEGG Pathways
Underrepresented
Overrepresented
−80
−40
0
Gene Count
Streptophyta
G
Carbon metabolism
Biosynthesis of amino acids
Ribosome
Biosynthesis of cofactors
Oxidative phosphorylation
Pyruvate metabolism
Glycolysis / Gluconeogenesis
2−Oxocarboxylic acid metabolism
Citrate cycle (TCA cycle)
Glyoxylate and dicarboxylate metabolism
Glycine, serine and threonine metabolism
Aminoacyl−tRNA biosynthesis
Valine, leucine and isoleucine degradation
Alanine, aspartate and glutamate metabolism
Carbon fixation by Calvin cycle
KEGG Pathways
Underrepresented
Overrepresented
−80
−60
−40
−20
0
Gene Count
Bacillota

Supplementary Figure 5: Top 15 KEGG pathways enriched in the top six phyla based on differential gene expression analysis from the 2023 dataset. The phyla include **A** *Microcystis*, **B** *Cyanobacteriota* (excluding *Microcystis*), **C** *Pseudomonadota*, **D** *Bacteroidota*, **E** *Actinomycetota*, **F** *Streptophyta* and **G** *Bacillota*. Each panel shows the number of overrepresented (red) and underrepresented (blue) genes in key metabolic and functional pathways. KEGG pathways are ranked by gene count. *Cyanobacteriota* was divided into *Microcystis* and non-*Microcystis Cyanobacteriota*.

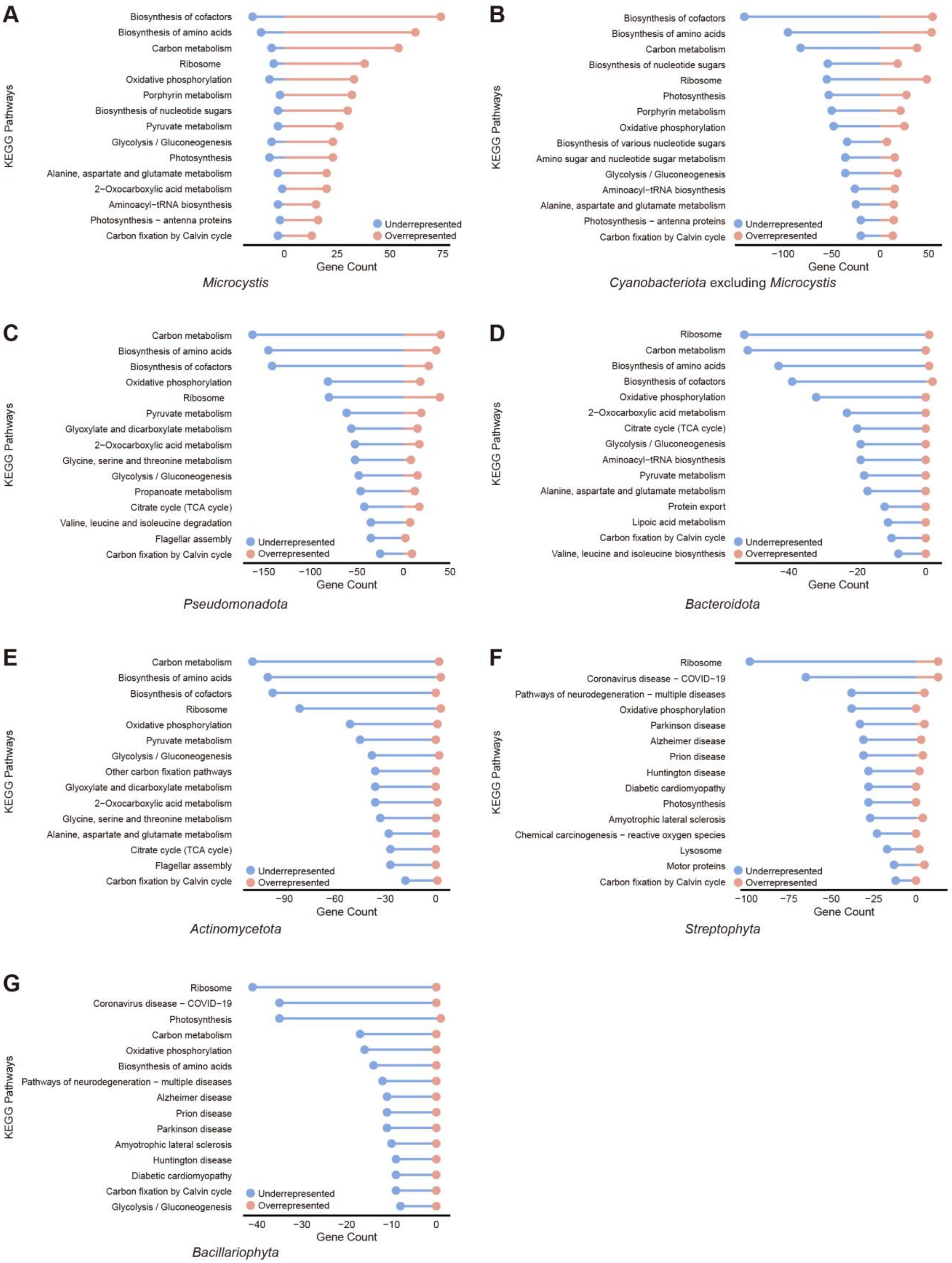
A
KEGG Pathways
Biosynthesis of cofactors
Biosynthesis of amino acids
Carbon metabolism
Ribosome
Oxidative phosphorylation
Porphyrin metabolism
Biosynthesis of nucleotide sugars
Pyruvate metabolism
Glycolysis / Gluconeogenesis
Photosynthesis
Alanine, aspartate and glutamate metabolism
2−Oxocarboxylic acid metabolism
Aminoacyl−tRNA biosynthesis
Photosynthesis − antenna proteins
Carbon fixation by Calvin cycle
Underrepresented
Overrepresented
0
25
50
75
Gene Count
Microcystis
B
KEGG Pathways
Biosynthesis of cofactors
Biosynthesis of amino acids
Carbon metabolism
Biosynthesis of nucleotide sugars
Ribosome
Photosynthesis
Porphyrin metabolism
Oxidative phosphorylation
Biosynthesis of various nucleotide sugars
Amino sugar and nucleotide sugar metabolism
Glycolysis / Gluconeogenesis
Aminoacyl−tRNA biosynthesis
Alanine, aspartate and glutamate metabolism
Photosynthesis − antenna proteins
Carbon fixation by Calvin cycle
Underrepresented
Overrepresented
−100
−50
0
50
Gene Count
Cyanobacteriota excluding Microcystis
C
KEGG Pathways
Carbon metabolism
Biosynthesis of amino acids
Biosynthesis of cofactors
Oxidative phosphorylation
Ribosome
Pyruvate metabolism
Glyoxylate and dicarboxylate metabolism
2−Oxocarboxylic acid metabolism
Glycine, serine and threonine metabolism
Glycolysis / Gluconeogenesis
Propanoate metabolism
Citrate cycle (TCA cycle)
Valine, leucine and isoleucine degradation
Flagellar assembly
Carbon fixation by Calvin cycle
Underrepresented
Overrepresented
−150
−100
−50
0
50
Gene Count
Pseudomonadota
D
KEGG Pathways
Ribosome
Carbon metabolism
Biosynthesis of amino acids
Biosynthesis of cofactors
Oxidative phosphorylation
2−Oxocarboxylic acid metabolism
Citrate cycle (TCA cycle)
Glycolysis / Gluconeogenesis
Aminoacyl−tRNA biosynthesis
Pyruvate metabolism
Alanine, aspartate and glutamate metabolism
Protein export
Lipoic acid metabolism
Carbon fixation by Calvin cycle
Valine, leucine and isoleucine biosynthesis
Underrepresented
Overrepresented
−40
−20
0
Gene Count
Bacteroidota
E
KEGG Pathways
Carbon metabolism
Biosynthesis of amino acids
Biosynthesis of cofactors
Ribosome
Oxidative phosphorylation
Pyruvate metabolism
Glycolysis / Gluconeogenesis
Other carbon fixation pathways
Glyoxylate and dicarboxylate metabolism
2−Oxocarboxylic acid metabolism
Glycine, serine and threonine metabolism
Alanine, aspartate and glutamate metabolism
Citrate cycle (TCA cycle)
Flagellar assembly
Carbon fixation by Calvin cycle
Underrepresented
Overrepresented
−90
−60
−30
0
Gene Count
Actinomycetota
F
KEGG Pathways
Ribosome
Coronavirus disease − COVID−19
Pathways of neurodegeneration − multiple diseases
Oxidative phosphorylation
Parkinson disease
Alzheimer disease
Prion disease
Huntington disease
Diabetic cardiomyopathy
Photosynthesis
Amyotrophic lateral sclerosis
Chemical carcinogenesis − reactive oxygen species
Lysosome
Motor proteins
Carbon fixation by Calvin cycle
Underrepresented
Overrepresented
−100
−75
−50
−25
0
Gene Count
Streptophyta
G
KEGG Pathways
Ribosome
Coronavirus disease − COVID−19
Photosynthesis
Carbon metabolism
Oxidative phosphorylation
Biosynthesis of amino acids
Pathways of neurodegeneration − multiple diseases
Alzheimer disease
Prion disease
Parkinson disease
Amyotrophic lateral sclerosis
Huntington disease
Diabetic cardiomyopathy
Carbon fixation by Calvin cycle
Glycolysis / Gluconeogenesis
Underrepresented
Overrepresented
−40
−30
−20
−10
0
Gene Count
Bacillariophyta

Supplementary Figure 6: Top 15 KEGG pathways enriched in the top six phyla based on differential gene expression analysis from the 2018 dataset. The phyla include **A** *Microcystis*, **B** *Cyanobacteriota* (excluding *Microcystis*), **C** *Pseudomonadota*, **D** *Bacteroidota*, **E** *Actinomycetota*, **F** *Streptophyta* and **G** *Bacillariophyta*. Each panel shows the number of overrepresented (red) and underrepresented (blue) genes in key metabolic and functional pathways. KEGG pathways are ranked by gene count. *Cyanobacteriota* was divided into *Microcystis* and non-*Microcystis Cyanobacteriota*.

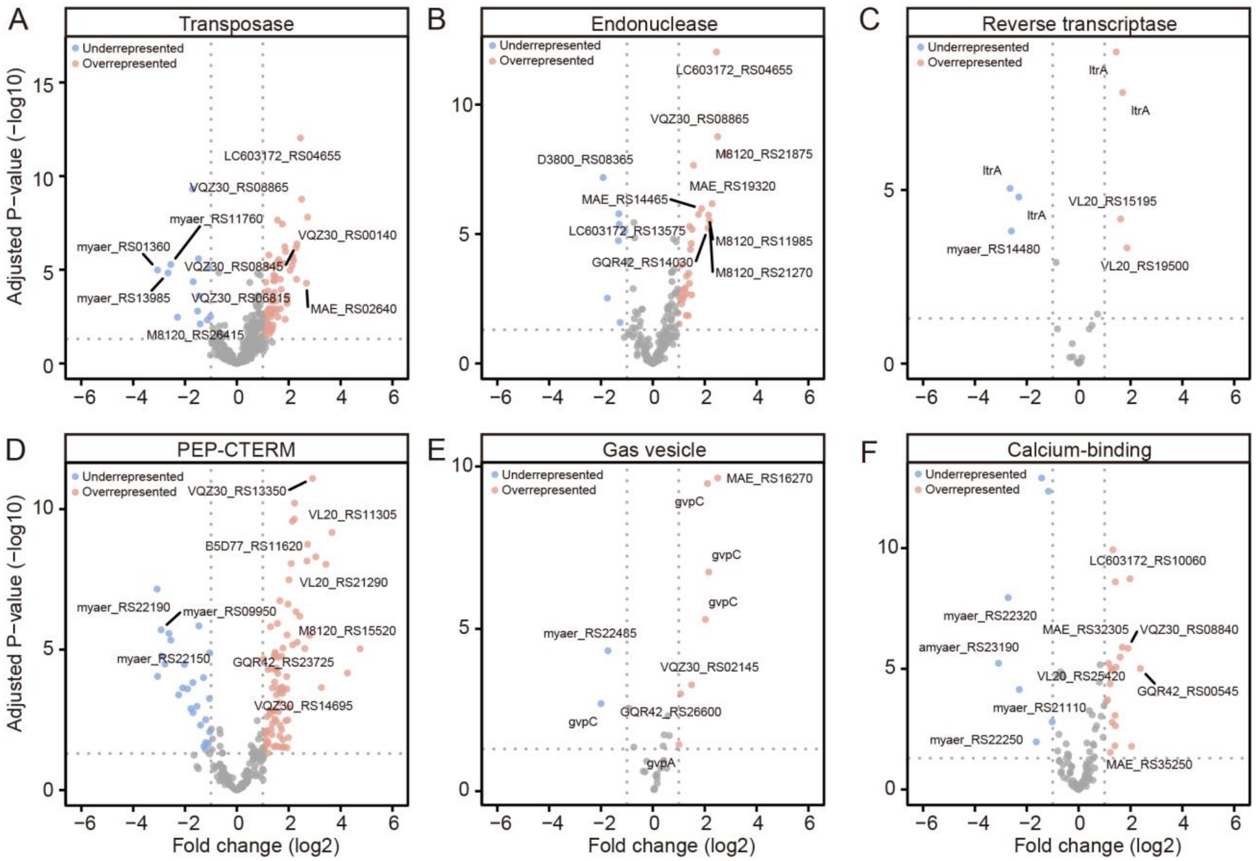

Supplementary Figure 7: Volcano plots depicting the differential expression and significance of genes across 2023 colonial and single-cell samples. **A**, **B**, and **C** highlight key genes categorized under COG category L, specifically transposase (**A**), endonuclease (**B**), and reverse transcriptase (**C**). **D**, **E**, and **F** present genes without COG classification, showcasing PEP-CTERM (**D**), gas vesicle proteins (**E**), and calcium-binding proteins (**F**). Red and blue points denote overrepresented and underrepresented genes, respectively, while gray points represent non-significant genes (adjusted *P*-value > 0.05). Annotated points indicate specific genes of interest.

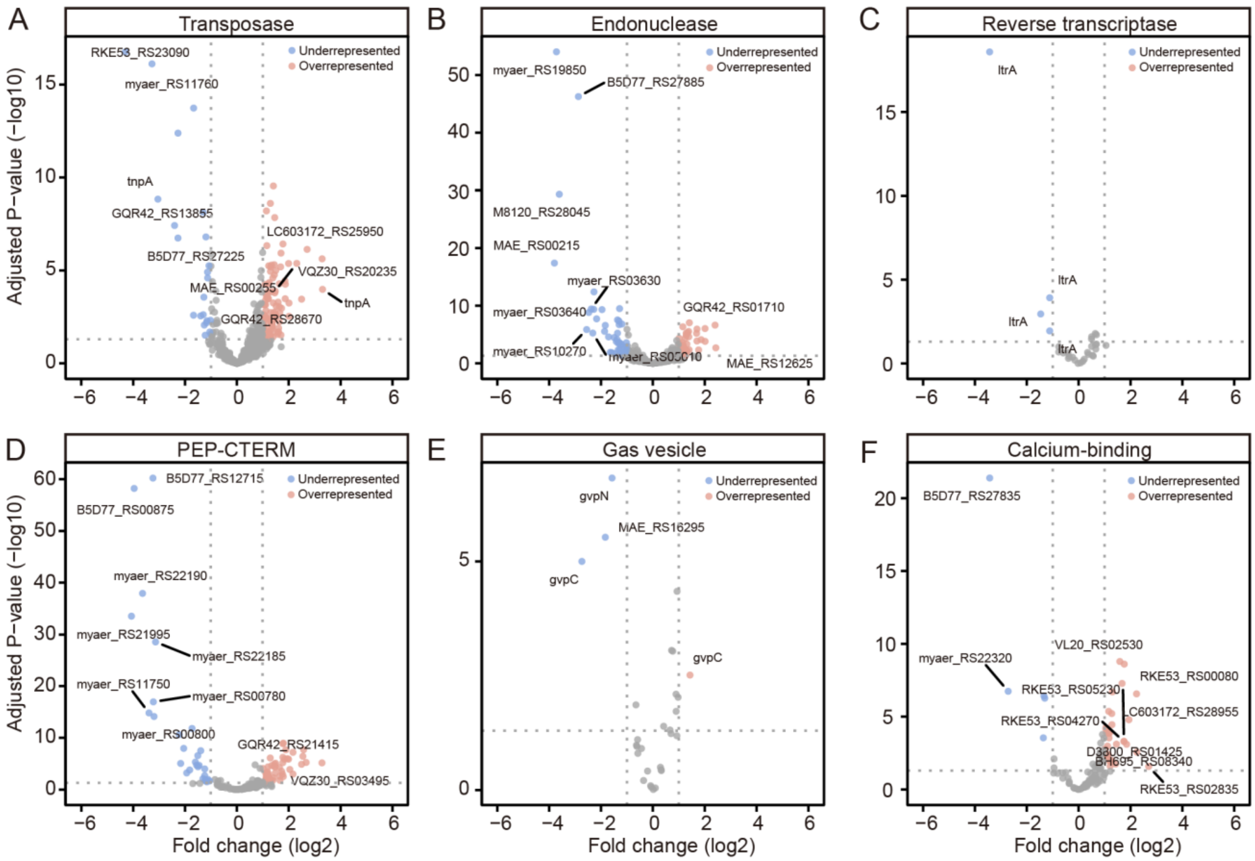

Supplementary Figure 8: Volcano plots depicting the differential expression and significance of genes across 2018 colonial and single-cell samples. **A**, **B**, and **C** highlight key genes categorized under COG category L, specifically transposase (**A**), endonuclease (**B**), and reverse transcriptase (**C**). **D**, **E**, and **F** present genes without COG classification, showcasing PEP-CTERM (**D**), gas vesicle proteins (**E**), and calcium-binding proteins (**F**). Red and blue points denote overrepresented and underrepresented genes, respectively, while gray points represent non-significant genes (adjusted *P*-value > 0.05). Annotated points indicate specific genes of interest.

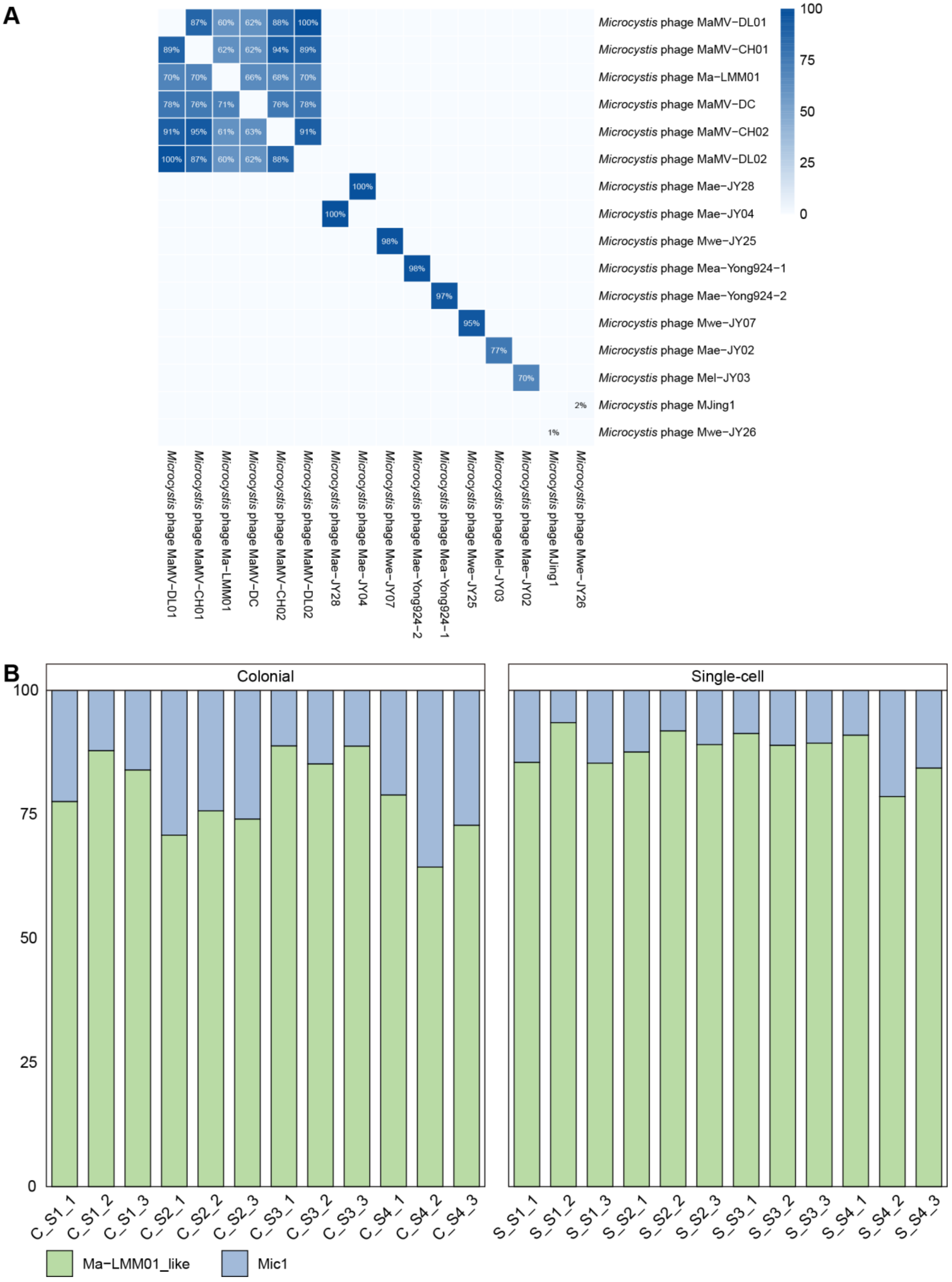

A
Microcystis phage MaMV−DL01
Microcystis phage MaMV−CH01
Microcystis phage Ma−LMM01
Microcystis phage MaMV−DC
Microcystis phage MaMV−CH02
Microcystis phage MaMV−DL02
Microcystis phage Mae−JY28
Microcystis phage Mae−JY04
Microcystis phage Mwe−JY25
Microcystis phage Mea−Yong924−1
Microcystis phage Mae−Yong924−2
Microcystis phage Mwe−JY07
Microcystis phage Mae−JY02
Microcystis phage Mel−JY03
Microcystis phage MJing1
Microcystis phage Mwe−JY26
100
75
50
25
0
B
Colonial
Single-cell
Relative abundance of Microcystis phage (%)
C_S1_1 C_S1_2 C_S1_3 C_S2_1 C_S2_2 C_S2_3 C_S3_1 C_S3_2 C_S3_3 C_S4_1 C_S4_2 C_S4_3
S_S1_1 S_S1_2 S_S1_3 S_S2_1 S_S2_2 S_S2_3 S_S3_1 S_S3_2 S_S3_3 S_S4_1 S_S4_2 S_S4_3
Ma−LMM01_like
Mic1

Supplementary Figure 9: Pangenome clustering and abundance distribution of *Microcystis* phages. **A,** Pangenome clustering of *Microcystis* phages. The heatmap shows pairwise gene cluster sharing between phage genomes. Percentages represent the proportion of shared clusters normalized by the total gene count of the phage genome on each row. **B,** Relative abundance of *Microcystis* phages in 2023 samples based on read mapping to the *Microcystis* phage pangenome. The Ma-LMM01-like group includes Ma-LMM01, MaMV-DC, MaMV-DL01, MaMV-DL02, MaMV-CH01, and MaMV-CH02.

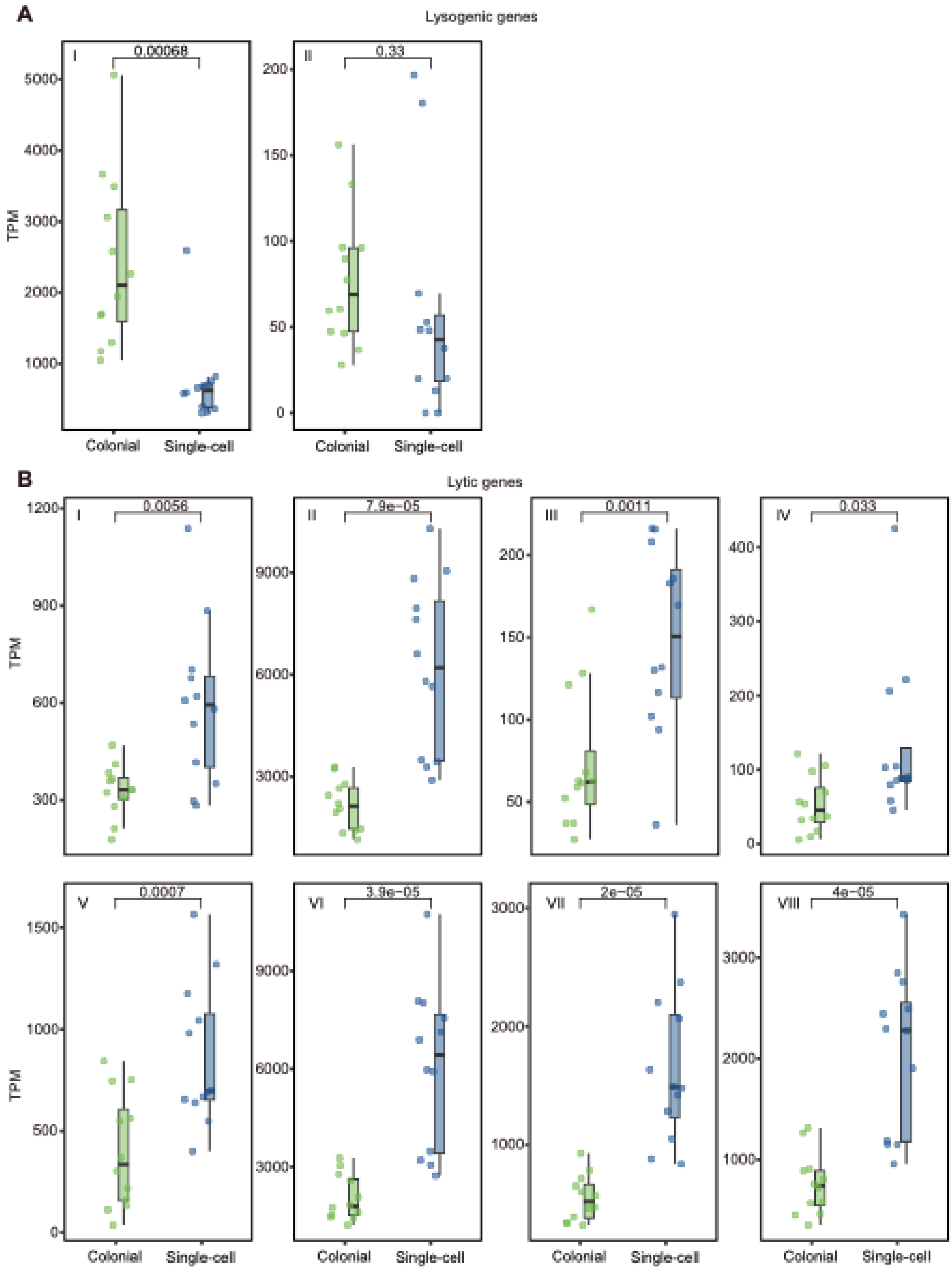
A
Lysogenic genes
I
0.00068
II
0.33
TPM
Colonial
Single-cell
B
Lytic genes
I
0.0056
II
7.9e−05
III
0.0011
IV
0.033
V
0.0007
VI
3.9e−05
VII
2e−05
VIII
4e−05
TPM
Colonial
Single-cell

Supplementary Figure 10: Expression of additionally selected lysogenic and lytic genes based on the 2023 database**. A.** Lysogenic genes (TPM values in colonial and single-cell *Microcystis*) include *I.* MaLMM01*_gp031* (transposase) and *II.* MaLMM01*_gp032* (transposase). **B.** Lytic genes (TPM values in colonial and single-cell *Microcystis*) include *I.* MaLMM01*_gp009* (anti-repressor), *II.* MaLMM01*_gp019* (tail collar protein), *III.* MaLMM01*_gp024* (anti-repressor Ant), *IV.* MaLMM01*_gp068* (endolysin), *V.* MaLMM01*_gp069* (endolysin), *VI.* MaLMM01*_gp083* (tail protein), *VII.* MaLMM01*_gp094* (baseplate protein), and *VIII.* MaLMM01*_gp106* (putative lysine/ornithine N-monooxygenase). Genes were selected from the *Microcystis* phage pangenome based on functional annotations containing the following keywords: lysogeny (integrase, repressor, excisionase, transposase) and lytic (anti-repressor, lysozyme, holin, endolysin, tail, capsid, portal, sheath, scaffold, baseplate). Genes *gp135*, *gp136*, and *gp091* were excluded as they are already shown in the main text.

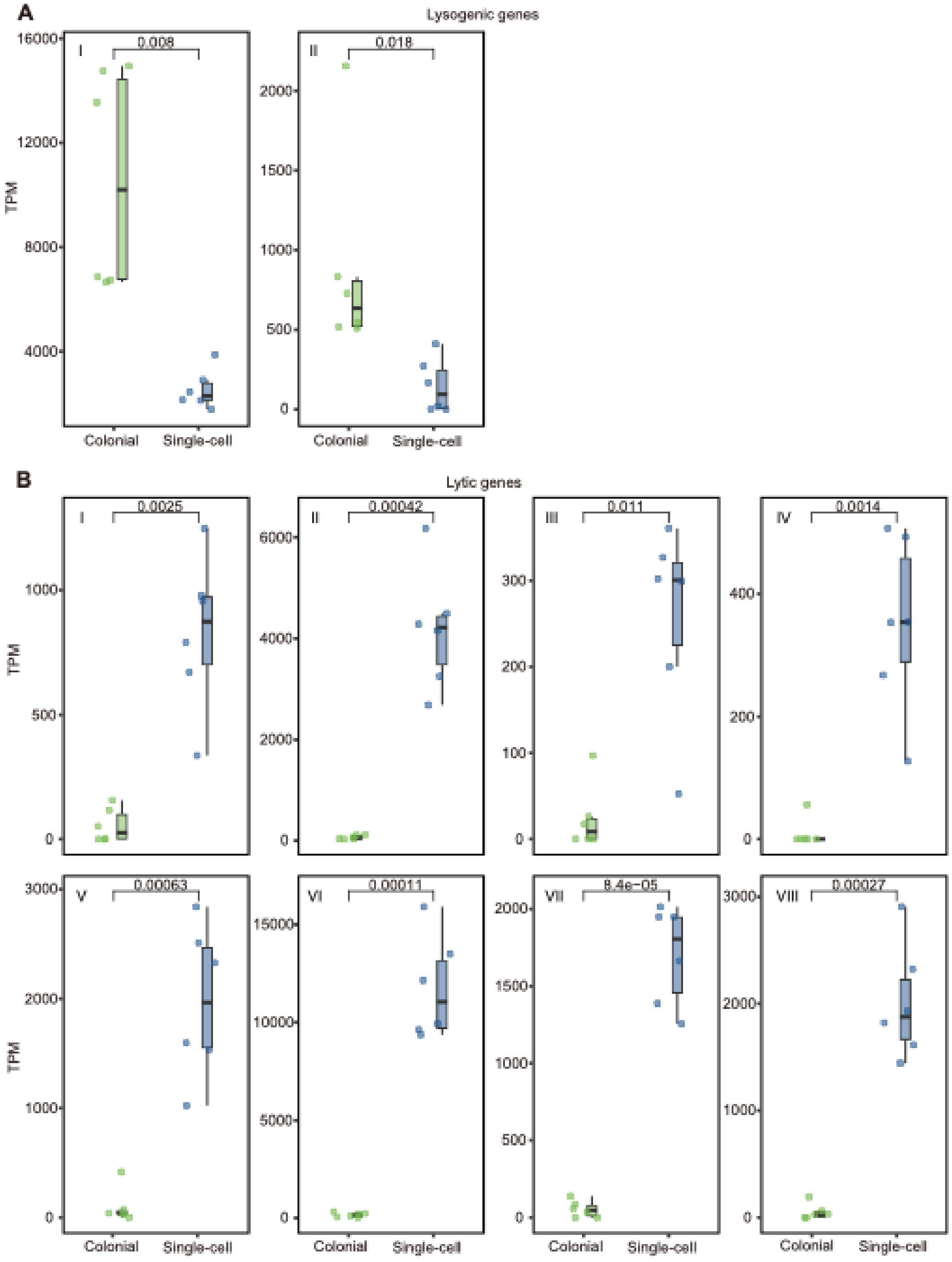

A
Lysogenic genes
I
0.008
II
0.018
TPM
Colonial
Single-cell
B
Lytic genes
I
0.0025
II
0.00042
III
0.011
IV
0.0014
V
0.00063
VI
0.00011
VII
8.4e-05
VIII
0.00027

Supplementary Figure 11: Expression of additionally selected lysogenic and lytic genes based on the 2018 database**. A.** Lysogenic genes (TPM values in colonial and single-cell *Microcystis*) include *I.* MaLMM01_*gp031* (transposase) and *II.* MaLMM01_*gp032* (transposase). **B.** Lytic genes (TPM values in colonial and single-cell *Microcystis*) include *I.* MaLMM01_*gp009* (anti-repressor), *II.* MaLMM01_*gp019* (tail collar protein), *III.* MaLMM01_*gp024* (anti-repressor Ant), *IV.* MaLMM01_*gp068* (endolysin), *V.* MaLMM01_*gp069* (endolysin), *VI.* MaLMM01_*gp083* (tail protein), *VII.* MaLMM01_*gp094* (baseplate protein), and *VIII.* MaLMM01_*gp106* (putative lysine/ornithine N-monooxygenase). Genes were selected from the *Microcystis* phage pangenome based on functional annotations containing the following keywords: lysogeny (integrase, repressor, excisionase, transposase) and lytic (anti-repressor, lysozyme, holin, endolysin, tail, capsid, portal, sheath, scaffold, baseplate). Genes *gp135*, *gp136*, and *gp091* were excluded as they are already shown in the main text.

References

1. Zepernick BN, Chase EE, Denison ER et al. Declines in ice cover are accompanied by light limitation responses and community change in freshwater diatoms. *ISME J* 2024;**18**:wrad015. https://doi.org/10.1093/ismejo/wrad015
